# Auto reference vector: a novel method for mapping atrial fibrillation

**DOI:** 10.3389/fcvm.2026.1719071

**Published:** 2026-03-02

**Authors:** Tetsuro Takase, Masahiro Ishikura, Akifumi Mitsushima, Akira Shinoda, Tasuku Morimoto, Yoshio Furukawa

**Affiliations:** 1 Department of Cardiology, Ichinomiya Nishi Hospital, Aichi, Japan; 2 Department of Clinical Engineering, Ichinomiya Nishi Hospital, Aichi, Japan

**Keywords:** atrial fibrillation, auto reference vector, catheter ablation, non-pulmonary vein foci, vector-based activation mapping

## Abstract

**Background:**

A standardized approach for constructing electroanatomical activation maps of atrial fibrillation (AF) has not yet been established.

**Objective:**

This study introduces a novel mapping technique, the Auto Reference Vector (ARV), and evaluates its ability to characterize AF activation patterns and guide ablation of focal sources, with a particular emphasis on achieving AF non-inducibility.

**Methods:**

Forty-seven patients with AF (mean age, 71.1 ± 11.6 year; 30 males; 30 with persistent AF; 34 undergoing repeat procedures) underwent catheter ablation. During AF mapping, the B2-C2 electrodes of a high-density grid catheter served as an internal reference to compute omnipolar vectors. Activation patterns were classified, and focal sources characterized by predominant centrifugal vectors were targeted for ablation. In 12 patients, repeated mapping was performed to assess the reproducibility of ARV data.

**Results:**

Nearly all redo cases (33/34) demonstrated organized activation whereas most *de novo* cases (9/13) showed disorganized patterns. Prior ablation and longer AF cycle length were significantly associated with organized activation (*p* < 0.001 and *p* = 0.02, respectively). In total, 113 focal sources were identified in 41 patients, the majority located in the left atrium (91/113; 80%). Ablation of these sources led to immediate AF termination in 12 of 38 patients (32%) and achieved final AF non-inducibility in 33 of 38 patients (87%).

**Conclusions:**

ARV mapping enables reliable identification of organized AF activation patterns, particularly in patients with prior ablation. Targeted ablation of ARV-identified focal sources effectively suppresses AF. Further studies are warranted to validate the long-term efficacy of this strategy.

## 1 Introduction

Pulmonary vein isolation (PVI) is widely accepted as the standard treatment for atrial fibrillation (AF). However, its efficacy remains limited, particularly in patients with persistent AF (PeAF) (1, 2). In cases where AF recurs despite successful PVI and without evidence of pulmonary vein reconnection, various adjunctive ablation strategies have been proposed. These approaches are generally categorized as either trigger-based or substrate-based strategies.

Trigger-based methods target premature atrial contractions that initiate AF episodes, whereas substrate-based approaches focus on modifying the arrhythmogenic atrial substrate. Examples of substrate modification techniques include low-voltage area ablation (2), ablation of fractionated atrial activity (3), empirical posterior wall isolation, focal impulse and rotor modulation–guided ablation (4), complex fractionated atrial electrogram ablation (5), CARTOFINDER mapping, and spatiotemporal dispersion mapping.

Despite the variety of available strategies, the STAR-AF II trial (6) has shifted clinical practice toward PVI-only approaches in PeAF patients, primarily due to the lack of reproducibility and consistent outcomes associated with substrate-based and trigger-based techniques. Key challenges in expanding ablation beyond PVI include an incomplete understanding of AF mechanisms, significant patient-to-patient variability, technical limitations in creating durable lesions, and the absence of standardized procedural endpoints.

A major unmet need in AF mapping is the ability to reconstruct reliable activation patterns during ongoing AF, which remains technically challenging because of the absence of a consistent reference signal, ambiguity in wavefront annotation, frequent wavefront collisions, and the highly dynamic nature of fibrillatory conduction.

In this study, we propose a novel three-dimensional (3D) electroanatomical mapping approach that utilizes the Auto Reference Vector (ARV) technique with omnipolar technology (OT). This method offers high spatial resolution and enables visualization of AF propagation without relying on external reference points. We hypothesize that ARV mapping can identify focal sources that sustain AF and serve as effective ablation targets, thereby improving procedural outcomes in patients with persistent or recurrent AF.

## 2 Methods

### 2.1 Study design and patient population

This prospective study enrolled 47 nonconsecutive patients with persistent AF who underwent catheter ablation at Ichinomiya Nishi Hospital between December 2024 and August 2025. The study protocol was approved by the Institutional Ethics Committee, and written informed consent was obtained from all participants.

Antiarrhythmic medications were discontinued before the procedure for a duration equivalent to at least four drug half-lives. All patients were maintained on direct oral anticoagulants for a minimum of 2 weeks before the procedure.

### 2.2 ARV mapping of AF

Following the induction of general anesthesia and transseptal puncture, 3D electroanatomical mapping was performed using the EnSite™ × EP System (Abbott, Abbott Park, Illinois) in conjunction with the Advisor™ HD Grid (HDG) or HD Grid X Mapping Catheter, Sensor Enabled™ (Abbott). Mapping was conducted during ongoing AF in the left atrium (LA) or both atria.

**Figure 1:**
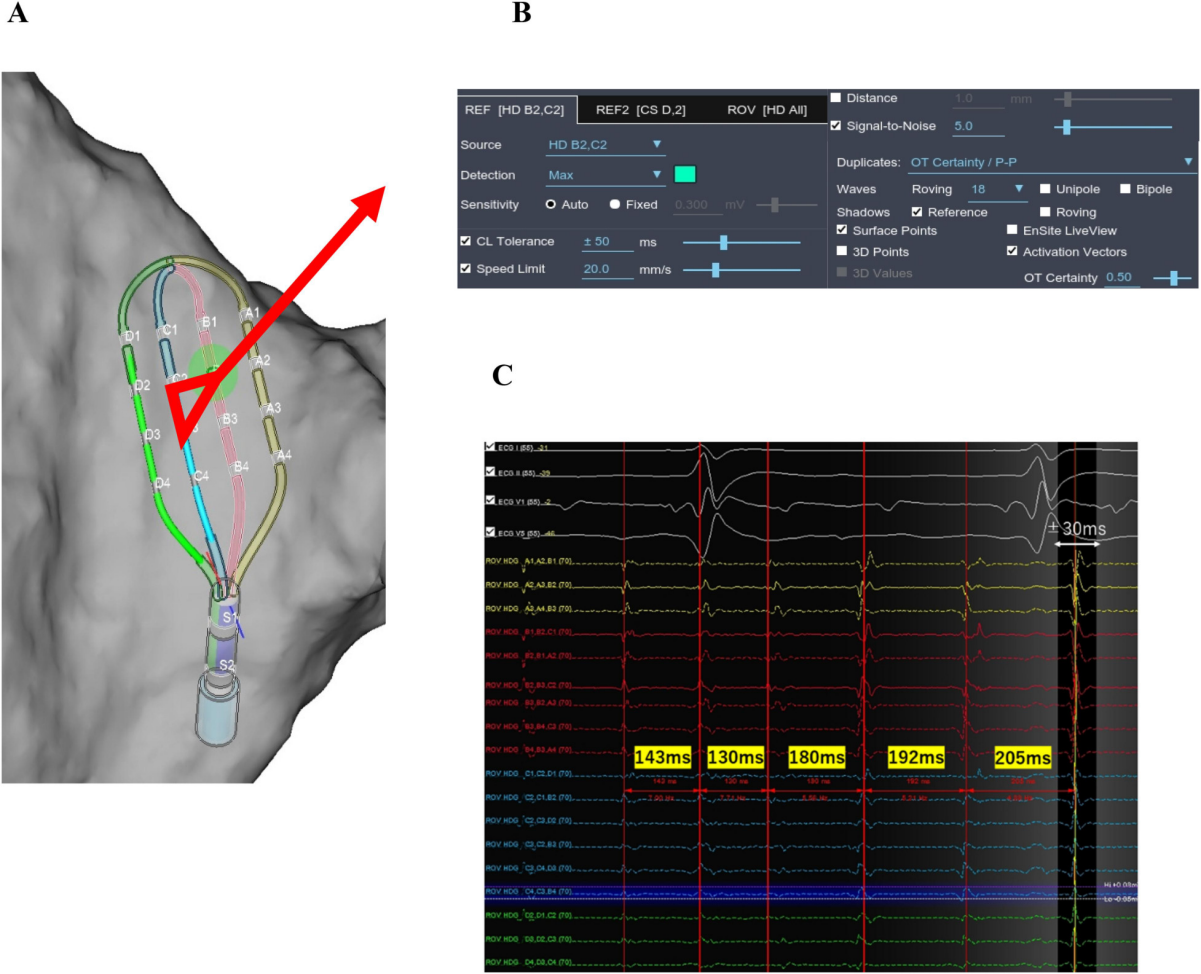
Auto reference Vector mapping configuration. **(A)** The omnipolar activation vector is calculated using the B2 and C2 electrodes as reference. **(B)** Reference electrode detection is set to maximum amplitude with auto-sensitivity. **(C)** The median AF cycle length is 180 ms. The cycle length tolerance is set to ±50 ms, and the window of interest is defined as ±30 ms from the reference annotation.

The ARV technique involved using electrodes B2 and C2 of the HD Grid catheter as self-referencing electrodes to generate omnipolar activation vectors. Reference annotations were initially set to peak amplitude with auto-sensitivity (Figure 1). The sensitivity setting was lowered to 0.02 mV in low atrial amplitude area. The mean AF cycle length (AFCL) was calculated by measuring ten consecutive electrograms recorded from catheters positioned in left and right atrial appendage (LAA, RAA). Cycle length tolerance was set to ±50 ms. The initial window of interest (WOI) was set to ±30 ms relative to the annotation at the reference electrodes and was widened if electrograms extended beyond the initial WOI.

To ensure reliable annotation, duplicate setting of OT certainty/ peak-to-peak (P-P) 0.5 (nominal 0.3) was applied. The score threshold was set to ≥0 to exclude ventricular annotations, which typically yield negative scores (Supplementary Figure S1).

This mapping configuration enabled continuous, real-time acquisition of omnipolar vectors throughout the atrial chambers without the need for prolonged catheter stabilization at specific sites (Supplementary Video 1).

After mapping of left atrium, we arbitrarily divided the whole chamber in to 18 segments. Each segment was considered organized if >70% of adjacent vectors were coherent in one direction. The chamber as a whole was considered organized if >70% (13/18) of segments were organized (Supplementary Figure S11).

To assess reproducibility, mapping was repeated in four patients immediately after the initial acquisition and in seven patients following cardioversion and AF reinduction. For each pair of maps, vector concordance was evaluated across the mapped area, and reproducibility was classified as low (<30%), moderate (30%–70%), or high (>70%).

**Figure 2:**
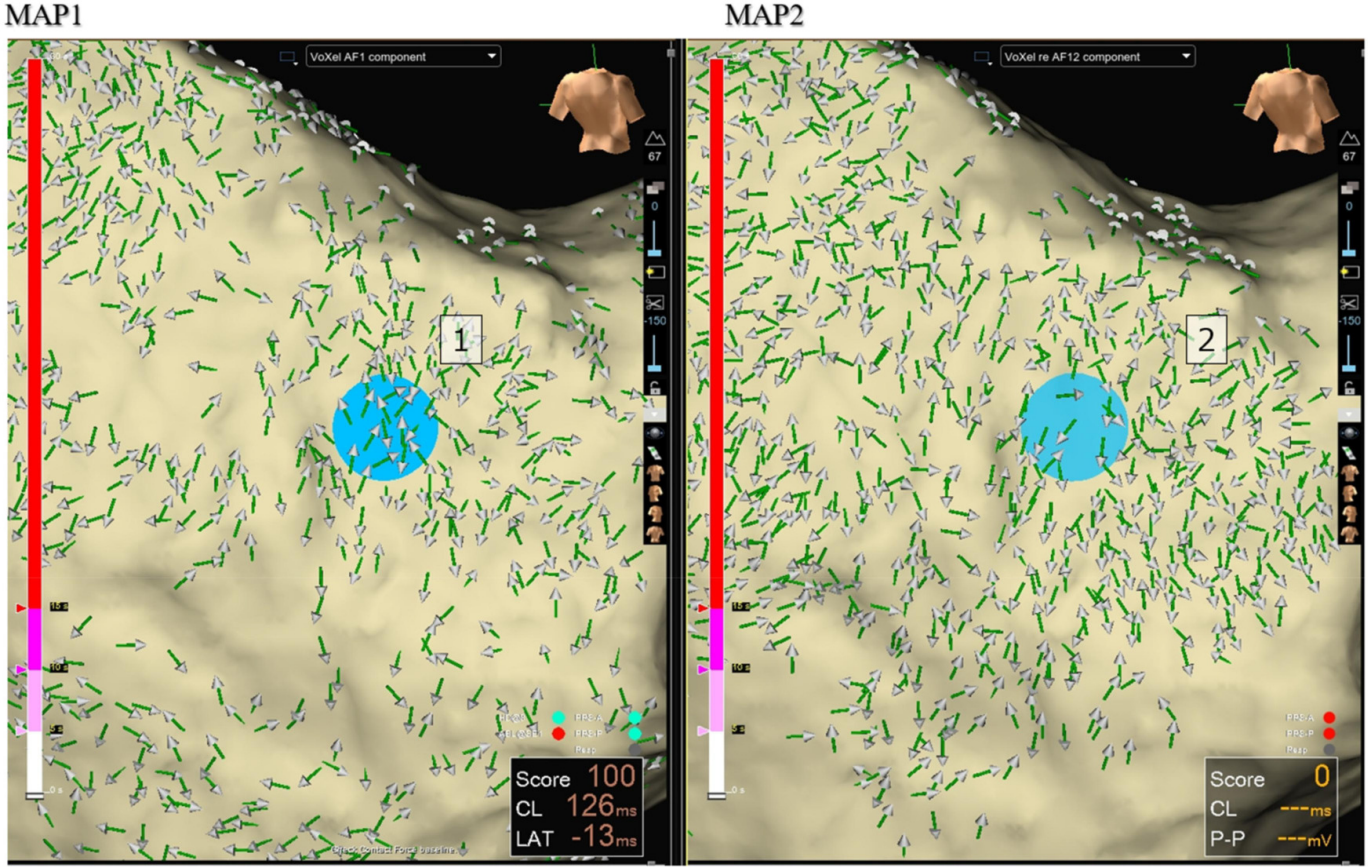
Disorganized activation pattern (CASE 1). ARV mapping (MAP1) of left atrium in a *de novo* case revealed a chaotic activation pattern. A repeat map (Map 2), performed immediately after Map 1, demonstrated poor reproducibility of vector directions. Blue dots indicate the reference point on the left atrial posterior wall, consistent across both maps.

To objectively identify focal sources of AF, Focal Vector Score (FVS) system was developed. A circular region of interest (approximately 0.8 × 0.8 cm^2^) surrounding the suspected centrifugal center was divided into eight equal sectors. Each sector was assigned a score: +1 for >60% centrifugal vectors, −1 for >60% centripetal vectors, and 0 for predominantly perpendicular or bidirectional vectors (Figure 2). Regions with a cumulative FVS of ≥3 were considered valid focal sources and were primarily selected as ablation targets.

### 2.3 Ablation strategy

Radiofrequency ablation was performed using an irrigated-tip catheter (TactiFlex™, Abbott) at a high-power setting of 50 W for durations of 8–15 s. In two cases (Case 4, 22), the PulseSelect™ Pulsed Field Ablation System (Medtronic, Minneapolis, Minnesota) was utilized.

In *de novo* cases, ablation was directed toward PVI and focal sources identified by ARV mapping. In redo cases, ablation targeted only the identified focal sources. When the distance between an ablated area and an anatomical structure or a PVI line was ≤12 mm, a linear ablation was performed to connect the two sites to prevent the formation of iatrogenic reentrant circuits.

If ablation did not result in AF termination, electrical cardioversion was performed, followed by intravenous administration of isoproterenol (0.01 mg). AF was then reinduced using rapid and decremental atrial pacing (300–200 ms), and a new ARV map was generated to identify any new focal sources. This process was repeated until AF was no longer inducible. If AF remained inducible after four ARV mappings within the same chamber, the procedure was considered unsuccessful.

In cases where atrial tachycardia (AT) was induced, mapping and ablation were performed accordingly. If frequent atrial ectopic beats were observed after sinus rhythm restoration, targeted ablation of these ectopic foci was performed.

### 2.4 Follow up

Atrial tachyarrhythmia recurrence was defined as any documented episode lasting ≥30 s and occurring after a 90-day blanking period. Scheduled follow-up included standard 12-lead electrocardiograms (3-min recordings) at 3, 6, 9, and 12 months after the index procedure. In addition, symptom-driven 1-week Holter monitoring was performed in patients reporting recurrent symptoms. For patients with implanted pacemakers or implantable cardioverter-defibrillators (ICDs), recurrence was assessed based on device-recorded atrial tachycardia (AT) or atrial fibrillation (AF) episodes. Continued prescription of antiarrhythmic drugs after the blanking period was discouraged.

### 2.5 Statistical analysis

Categorical variables were reported as frequencies and percentages and compared using Pearson's chi-square test. Continuous variables were expressed as mean ± standard deviation (*SD*) and compared using Student's *t* test. All statistical analyses were two-tailed, and a *p* value <0.05 was considered statistically significant. Statistical computations were performed using Microsoft Excel 2019.

## 3 Result

### 3.1 Baseline clinical characteristics

A total of 47 patients with a mean age of 71.1 ± 11.6 years were enrolled, including 17 patients with paroxysmal and 30 with persistent AF (mean AF duration, 18.6 ± 36.0 months). Of these, 34 patients presented for repeat ablation due to AF recurrence, and 13 underwent *de novo* ablation. The mean left atrial volume index was 43.2 ± 15.9 mL/m^2^, and the mean left ventricular ejection fraction was 59.9 ± 7.9%. One patient underwent mitral valve repaired (Case 25), and another patient underwent mitral valve replacement combined with a Maze procedure (Case 30). Patient characteristics and procedural details are summarized in Table 1; Supplementary Table S1.

**Table 1:** Clinical characteristics at baseline.

Variable	*De novo* (*n* = 13)	redo (*n* = 34)	Total (*n* = 47)	*P* value
Age, y	66.0 ± 10.1	73.0 ± 11.5	70.8 ± 11.7	0.039
Sex, male	11 (84.6)	19 (55.9)	29 (64.4)	0.067
Body mass index	25.7 ± 4.0	24.9 ± 4.7	25.4 ± 46	0.503
Hypertension	5 (46.2)	19 (55.9)	24 (53.3)	0.285
Diabetes	2 (15.4)	5 (14.7)	7 (15.6)	0.953
Congestive heart failure	1 (7.7)	9 (26.5)	10 (22.2)	0.159
History of CVA/TIA	0 (0)	2 (5.9)	2 (4.4)	0.371
Type of atrial fibrillation				0.029
Paroxysmal	1 (7.7)	16 (47.1)	17 (37.8)	
Persistent	7 (53.8)	13 (38.2)	20 (44.4)	
Long-standing	5 (38.5)	5 (14.7)	8 (17.8)	
No. of previous ablation	0	1.8 ± 1.2	1.4 ± 1.3	<0.001
CHA2DS2VASC	1.8 ± 1.9	3.0 ± 1.5	2.6 ± 1.7	0.013
LVEF, %	60.5 ± 5.0	59.6 ± 8.8	60.1 ± 8.0	0.708
Left atrial volume index, mL/m^2^	40.2 ± 16.6	44.6 ± 15.2	43.1 ± 0.4	0.402

Values are mean ± SD or *n* (%). CVA, cerebrovascular accident; TIA, transient ischemic attack; LVEF, left ventricular ejection fraction.

### 3.2 ARV mapping

A total of 351,556 mapping points were acquired from 114 maps across 47 patients. The mean mapping durations were 22.3 ± 7.6 min for the first LA map, 14.0 ± 4.9 min for the second LA map, and 13.3 ± 5.6 min for the RA map. The average number of used points was 4,372 ± 1,403 (first LA map), 3,198 ± 1,194 (second LA map), and 3,372 ± 1,193 (RA map). The mean AFCL for RAA and LAA were 173.1 ± 27.3 and 172.2 ± 29.5 ms, respectively. Following focal source ablation, cycle length significantly prolonged in the RAA (194.5 ± 38.8 ms, *p* = 0.017) but not in the LAA (183.3 ± 29.1 ms, *p* = 0.113) (Supplementary Table S3).

**Figure 3:**
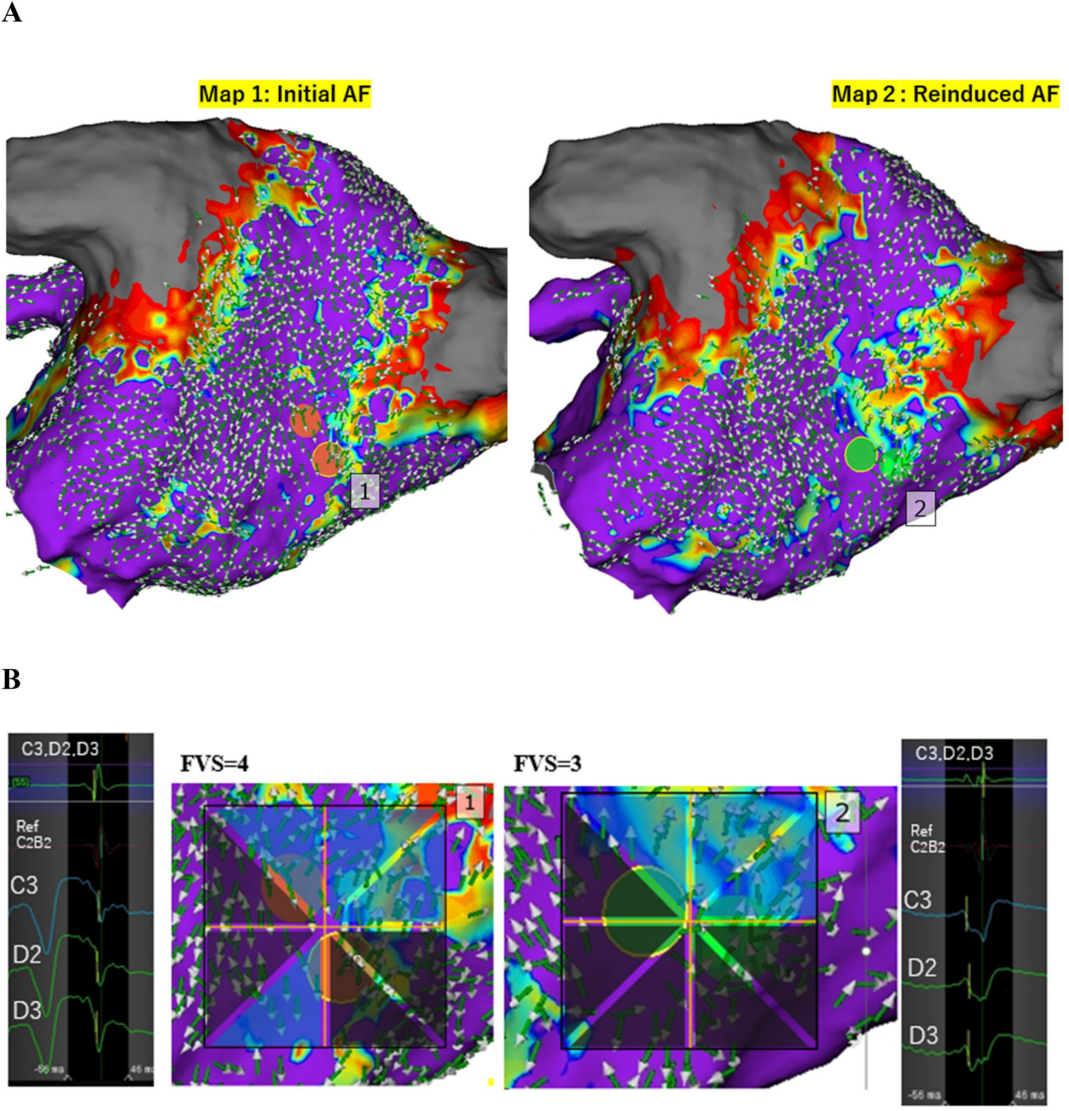
Organized activation pattern and reproducibility of focal sources. (CASE 6) **(A)** ARV mapping of the left atrium in a case with high reproducibility. AF spontaneously terminated due to mechanical stimulation from the HD Grid catheter near the location of the orange dots during Map 1 acquisition. AF was reinduced, and Map 2 was then created. Both maps demonstrated highly concordant ARV directions with a consistent collision pattern near the mid-posterior wall. Focal sources were repeatedly identified at the same location near the bottom of the previously isolated right inferior pulmonary vein (orange dots in Map 1 and green dot in Map 2). **(B)** The Focal vector score (FVS) values were 4 and 3 for Map 1 and Map 2, respectively. Both focal sources exhibited a QS pattern in unipolar electrograms.

In *de novo* cases, 70% showed disorganized patterns with low reproducibility (Figure 2), whereas 30% (4 cases) demonstrated organized, highly reproducible patterns (Supplementary Figure S4). By contrast, nearly all redo cases (97%) exhibited organized, highly reproducible patterns (Figure 3, Central illustration).

**Central Illustration:**
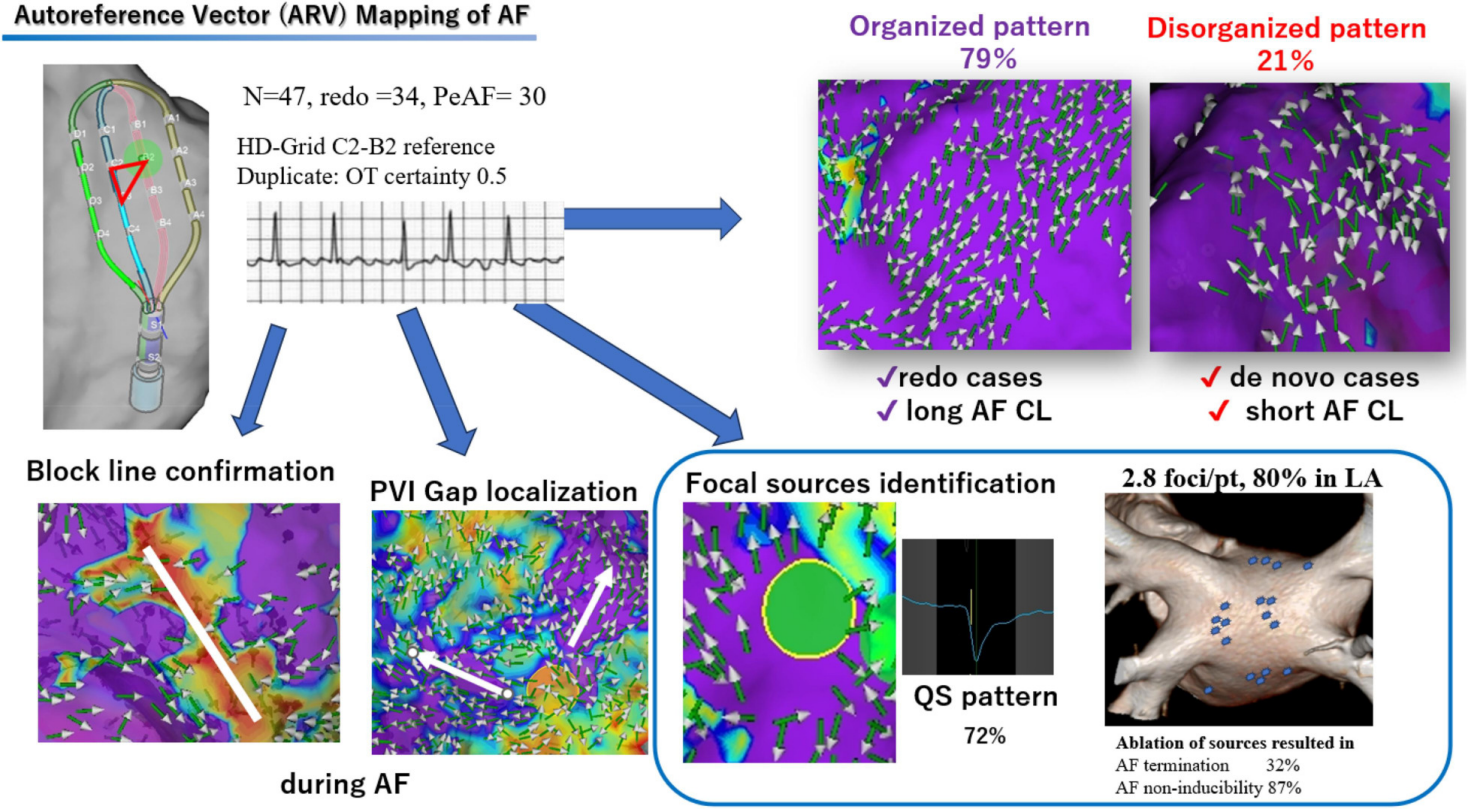
Mapping AF with the auto reference vector (ARV). ARV mapping enables categorization of AF activation patterns into organized and disorganized types. Prior ablation and longer AF cycle lengths are associated with increased AF organization. ARV mapping also facilitates confirmation of conduction block lines, identification of PVI gaps, and localization of focal sources acting as AF drivers. Ablation of these focal sources was associated with suppression of AT/AF. AF, atrial fibrillation; AT, atrial tachycardia; LA, left atrium; PVI, pulmonary vein isolation; CL, cycle length.

**Figure 4:**
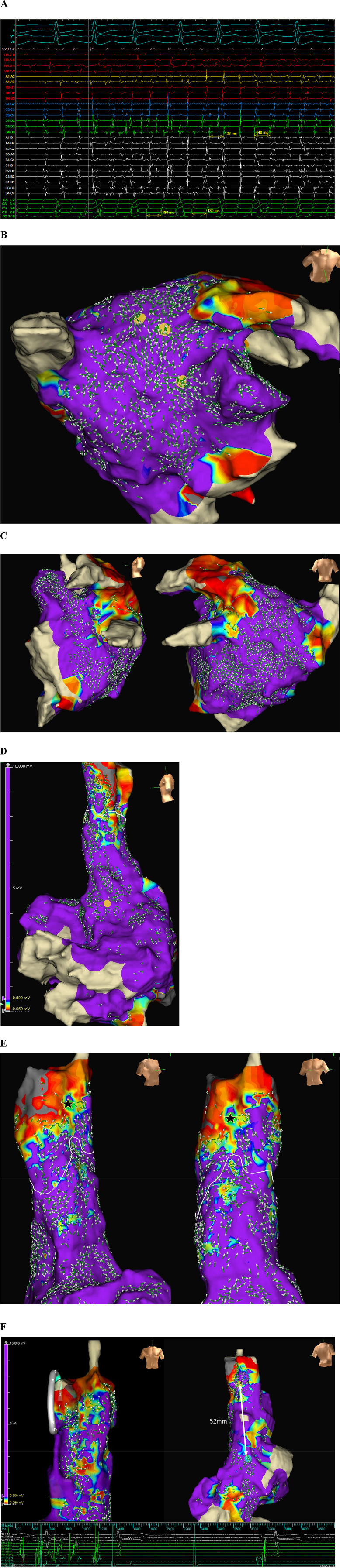
Representative case for ARV focal sources identified in both left and right atria (CASE 22). **(A)** The intracardiac electrograms showing AF with median cycle lengths of 140 ms and 132 ms measured at SVC and CS, respectively (HD grid positioned in SVC). **(B)** LA map showed an organized activation pattern with two focal sources near the LA roof and one near the LAA (yellow circles). **(C)** Others regions of the LA were passively activated, with vectors colliding at the posterior wall. **(D)** RA map showed passive conduction from Bachmann bundle (yellow circle). **(E)** Another focal source was identified in the anterolateral aspect of distal SVC (black star). A white line indicates the collision of activation vectors originating from SVC and those passively conducted from LA. **(F)** Ablation of three LA sources and one SVC source using a circular PFA catheter resulted in immediate termination of AF. The SVC source (yellow circle) was located 52 mm distal to the sinus node (cyan circle). Isoproterenol administration and repeated atrial decremental pacing failed to induce AT/AF afterwards. TCL, tachycardia cycle length; SVC, superior vena cava; CS, coronary sinus; LAA, left atrial appendage; PFA, pulsed field ablation.

ARV maps displaying broad centrifugal activation from the interatrial septum suggested either intrinsic septal focal sources or passive activation from the opposite chamber. Therefore, subsequent mapping of the opposite chamber is warranted (Figure 4; Supplementary Figure S5).

In Case 6, AF terminated after mechanical stimulation of a focal site near the right inferior pulmonary vein by the HD Grid catheter. Upon reinduction of AF, a nearly identical focal pattern was observed, with a centrifugal pattern originating near the base of the previously isolated right pulmonary vein and vector collision at the mid-posterior wall (Figure 3A).

In Case 9, extensive LA low voltage after multiple prior ablations resulted in reference annotation errors caused by diminished atrial signal amplitude. At the initial auto-sensitivity setting, small atrial potentials near the lateral mitral annulus were not detected, and large ventricular signals were incorrectly annotated instead. These points were subsequently excluded by negative scoring (Supplementary Video 1). After Adjusting the sensitivity to 0.01, a focal source in the same region was successfully identified (Supplementary Figure S6).

### 3.3 Focal source ablation and clinical outcomes

In total, 113 focal sources were identified by the subsequent ARV maps. The distribution of these sources is shown in Table 2: LA posterior region: 24 (21%), LA anterior region: 10 (8%), LA lateral region: 29 (26%), LA septal region: 10 (9%), LA roof region: 14 (12%) and RA region: 22 (19%).

**Table 2:** Spatial distribution of ARV-identified focal sources in the both atria.

Chamber	Location	No. of sources
LA (*n* = 91)	Anterior wall	10
	Posterior wall (PV level)	12
	Posterior wall (near RIPV bottom)	7
	Posterior wall (bottom)	5
	Roof	14
	LAA	7
	Ridge	8
	Lateral mitral isthmus	13
	GCV	1
	anterior septum	5
	Bachmann's bundle region	2
	RPV antrum	3
	LPV antrum	4
RA (*n* = 22)	RAA	6
	SVC	5
	Septal	4
	Lateral	3
	Anterior	2
	Posterior	2

No, number; LA, left atrium; RA, right atrium; GCV, great cardiac vein; RIPV, right inferior pulomonary vein; LAA, left atrial appendage; RAA, right atrial appendage; RPV, right pulmonary vein; LPV, left pulomary vein; SVC, superior vena cava.

At least one focal source was found in 41 (87%) out of 47 patients. Additionally, 71.7% (81/113) of all focal sites exhibited a QS pattern on unipolar electrogram recordings (Figure 3B; Supplementary Table S4).

Focal source locations overlapped with areas of high fractionation (1-s fractionation score >70%) in some patients (Case 4, Supplementary Figure S2), while in others, no fractionated potentials were observed near the focal sources. For example, in Case 6, fractionated signals were present at the LA posterior wall, but ARV analysis indicated passive activation from vector collision rather than a true focal source (Supplementary Figure S7). No correlation was found between focal sources and regions of high peak frequency (Supplementary Figure S3).

Following focal source ablation, 12 out of 38 patients (31.6%) spontaneously converted to sinus rhythm. Intracardiac cardioversion (10–15 J) was used in the remaining cases to terminate persistent AF. AT was induced in 19 patients and was mapped and ablated accordingly (Table 3). Among 38 patients in whom focal sources were identified and ablated, after administration of 0.01 mg of isoproterenol and a rigorous stimulation protocol, 33 (86.8%) of the patients demonstrated AF inducibility.

**Table 3:** Mapping and ablation outcomes.

Outcome	*de novo* (*n* = 13)	redo (*n* = 34)	*p* value
Organized pattern	4 (30.8)	33 (97.1)	<0.001
Mapping time(min)	34.1 ± 15.0	63.8 ± 68.3	0.135
Ablation time (min)	20.0 ± 9.6	16.6 ± 8.1	0.228
Total Procedural time (min)	155.8 ± 69.0	191.8 ± 53.8	0.071
AFL/AT induced during procedure	5 (38.4)	14 (41.2)	0.865
Patient with at least one focal source	7 (53.8)	33 (97.1)	<0.001
Identified focal sources/pt	1.57	3.09	0.076
Ablation targets			
ARV-identified focal sources	5	33	
PVI	13	1	
PV reisolation	0	4	
Roof line	1	5	
Posterior wall isolation	3	6	
Lateral mitral isthmus line	3	5	
SVCI	1	7	
CTI	3	8	
others	1	6	
AF termination during focal source ABL	2/5 (40.0)	10/33 (30.3)	0.664
AF/AT non-inducibility after focal source ABL	5/5 (100)	28/33 (84.8)	0.350
Final AF/AT non-inducibility in all pt	13/13 (100)	29/34 (85.3)	0.144
ARV map reproducibility (*n* = 12)^a^			
high	4/8 (50)	4/4 (100)	
low	4/8 (50)	–	

Values are mean ± SD or *n* (%).

PT, patient; AF, atrial fibrillation; AFL, atrial flutter; AT, atrial tachycardia; ARV, auto reference vector; PVI, pulmonary vein isolation; PV, pulmonary vein; SVCI, superior vena cava isolation; CTI, cavotricuspid isthmus; ABL, ablation.

a Reproducibility was tested only in a subset of 12 patients.

Case 14, one of four patients who demonstrated an organized activation pattern despite no prior PVI, was selected to assess the efficacy of ARV-guided focal ablation without PVI. Initial ARV mapping identified two focal sources at the LA roof and mitral isthmus. Following ablation and cardioversion, a second ARV map revealed three additional focal sources. Sequential ablation of all source organized AF into a roof-dependent AT., which was terminated by creation of a roofline block. AF was no longer inducible after repeated induction attempts, even without PVI. Nevertheless, PVI was performed as a precautionary measure at the end of the procedure (Supplementary Figure S8).

**Figure 5:**
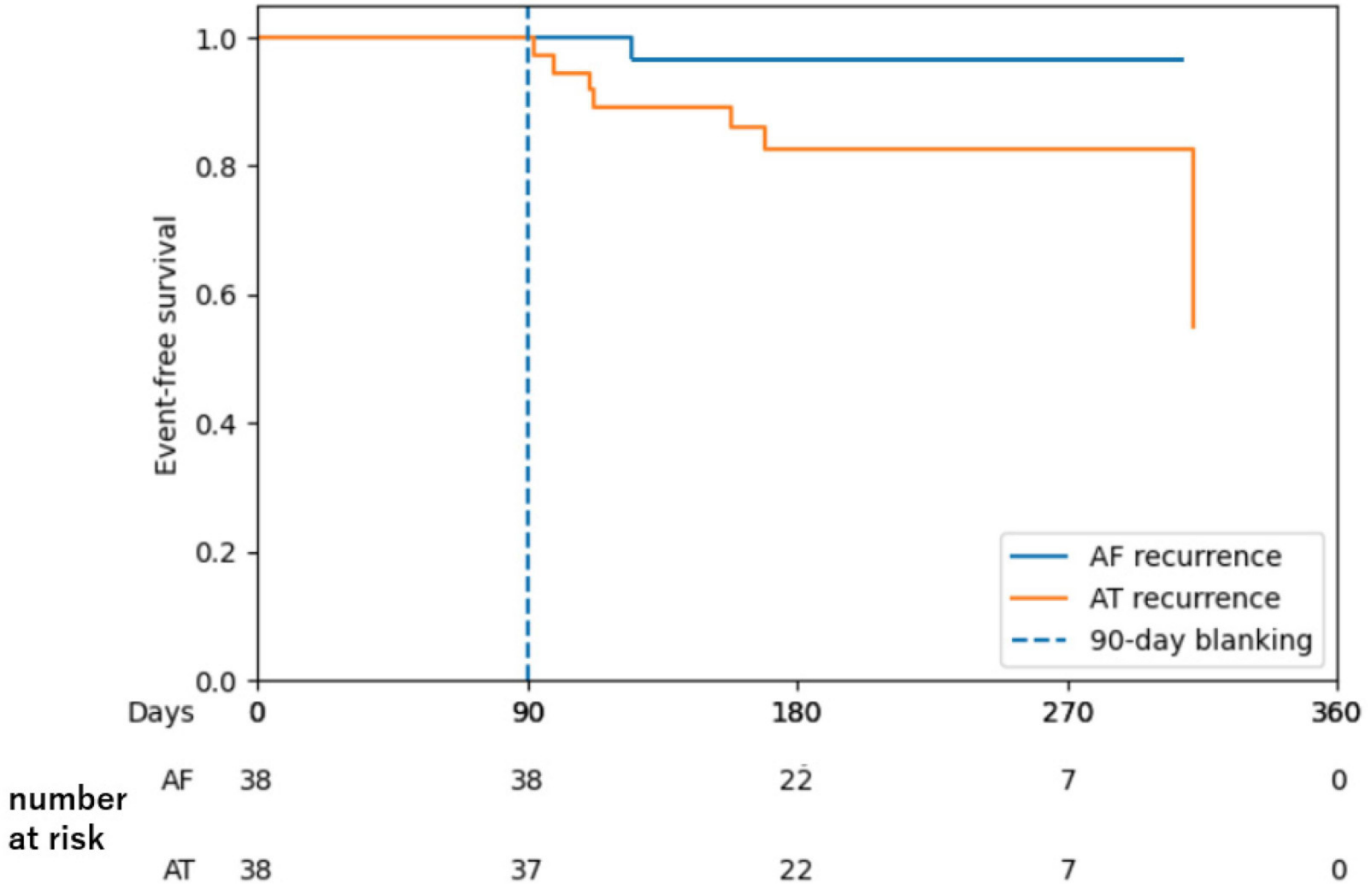
Kaplan–Meier curve showing atrial tachyarrhythmia–free survival in patients who underwent ablation of ARV-identified focal sources.

No cases of cardiac tamponade, stroke, esophageal fistula, or access-site related complications occurred in this cohort. The AT/AF-free survival of patients who underwent ablation of ARV-identified focal sources after a mean follow-up of 217 ± 65 days is shown in Figure 5.

## 4 Discussion

### 4.1 Main findings

This is the first prospective study to use an ARV approach to evaluate atrial activation patterns during AF. ARV mapping enables high-resolution visualization of AF propagation across both atria and identifies focal sources sustaining AF. Ablation of these focal sources led to AF termination in 31% and non-inducibility in 87% of the cases.

### 4.2 Mapping of AF with the ARV

The outcomes of a PVI-alone strategy remain suboptimal for patients with PeAF. Identifying and ablating non-PV sources have been associated with improved outcomes (7). However, accurate identification of these sources is challenging due to the instability of cycle length and wave front direction in AF.

Auto Reference Vector (ARV) with HDG presents a novel method for mapping AF, supplementing or improving upon existing techniques such as electrographic flow mapping (7), charge density mapping (8), and cycle length gradient mapping (9). Unlike traditional methods that depend on external reference electrodes (e.g., in the coronary sinus or appendages), ARV utilizes self-referencing based on HDG electrodes, improving spatial resolution and avoiding the inconsistencies of beat-to-beat variation.

The idea of using a roving reference point on the mapping catheter itself may initially seem counterintuitive, as it appears to introduce another element of instability, making it unclear how a cohesive activation map could be constructed. However, the ARV algorithm calculates local activation time (LAT) vectors for each beat independently and subsequently aligns them, much like assembling puzzle pieces, to identify consistent focal activation patterns throughout the chamber. This process is facilitated by Omnipolar technology (OT).

This process is facilitated by Omnipolar Technology (OT). OT determines the most reliable local activation vector by integrating unipolar and bipolar electrograms from a clique composed of three electrodes. OT certainty is a numeric confidence score ranging from 0 to 1 that reflects the trustworthiness of the computed local activation vector at each omnipolar point. Increasing the OT certainty threshold to 0.5 improved vector reliability by preferentially selecting high-voltage, directionally consistent signals while filtering out noise and contradictory vector orientations.

### 4.3 Mechanistic plausibility and signal validity

ARV-derived vectors represent beat-resolved local propagation; however, vector reliability is expected to degrade in regions of low signal-to-noise ratio, wavefront collision, or fractionation. We therefore applied an OT certainty threshold to preferentially retain directionally consistent signals and reduce contradictory vectors. The resulting maps preserved coherent propagation domains while suppressing low-confidence regions, supporting that the observed organized patterns are not solely driven by visualization artifacts but reflect stable local propagation (Supplementary Figure S12).

### 4.4 Interpretation of ARV

Organized activation patterns were observed in nearly all redo cases (33/34, 97%) but in only 30% of *de novo* cases (4/13) (Table 3), suggesting that prior PVI facilitates atrial organization. Longer AFCL also correlated with more organized activation (Table 4). These observations are consistent with mechanistic studies showing that pulmonary vein sleeves often harbor focal discharges or reentrant rotors with shorter cycle lengths than the surrounding atrium (10, 11).

**Table 4:** Factors affecting the organization of the AF pattern on ARV mapping.

Variable	Organized	Disorganized	*p* value
	(*n* = 36)	(*n* = 11)	
Prior PVI	33 (92%)	1 (9.0%)	<0.001
PAF	16 (44%)	0 (0%)	0.033
Male	22 (61%)	8 (72.7%)	0.483
Age (year)	72.4 ± 11.6	66.9 ± 10.5	0.180
LVEF (%)	59.3 ± 8.5	61.5 ± 5.5	0.429
LAD (mm)	42.4 ± 6.3	40.7 ± 6.1	0.444
LAVI (mL/m^2^)	45.2 ± 15.9	37.7 ± 14.0	0.177
BMI	25.3 ± 4.9	24.7 ± 3.0	0.751
LAA mean CL (ms)	177.5 ± 30.3	153.9 ± 18.4	0.021
RAA mean CL (ms)	176.8 ± 26.5	146.2 ± 9.7	0.011

Data are presented as mean ± SD or *n* (%).

LAD, left atrial diameter; LAVI, Left atrial volume index; LAA, left atrial appendage; RAA, right atrial appendage; CL, AF cycle length.

In the presence of intact PV sleeves, the AFCL is short and ARV mapping typically revealed highly disorganized patterns with multiple high-frequency colliding wavefronts and poor reproducibility. Isolation of these drivers removed the dominant source, resulting in slower, more organized wavefronts and occasionally conversion of AF to AT or sinus rhythm.

In patients with organized activation patterns, sequential mapping performed after AF termination and subsequent reinduction consistently reproduced near-identical activation, indicating that the focal sources were spatiotemporally stable and likely anchored to fixed structural substrates rather than transient phenomena. These observations challenge the conventional view of AF as purely chaotic and instead support the presence of organized, reproducible activation domains, particularly in patients with a history of PVI.

### 4.5 Interpretation of focal sources determined by ARV

In the present study, spontaneous activation originating from the ARV-identified focal source in the superior vena cava (SVC) triggered AF in three cases (e.g., Case 22, Figure 4). This finding suggests that, in specific cases, these focal sources may function not only as primary initiators but also as critical substrates or drivers sustaining AF.

Although interpretation of complex vectors in ARV maps and localization of ablation targets can be challenging when multiple centrifugal focal sources coexist, identification and exclusion of passively activated regions provide substantial clinical value. This information allows operators to avoid unnecessary ablation in non-contributory areas, thereby preserving atrial function and minimizing the risk of creating new arrhythmogenic substrates.

Furthermore, ARV enabled us to identify lines of conduction block even during ongoing AF (Supplementary Figure S9). Vector collision or termination was observed in all patients with prior ablation lines at the cavotricuspid isthmus, SVC, or LA roof. Additionally, ARV facilitated the identification of reconnections or gaps following PVI (Supplementary Figure S10).

Mechanistic validation of ARV mapping is supported by several observations: (1) reproducible focal activation patterns following AF termination and reinduction, indicating spatiotemporal stability; (2) correspondence of ARV-identified sources with spontaneous AF triggers in selected cases; and (3) use of omnipolar technology, which has been shown to more accurately delineate atrial scar during AF compared with bipolar mapping. Collectively, these findings support that ARV-defined focal sources reflect substrate-anchored drivers rather than transient wavefront phenomena.

### 4.6 Procedural endpoint

AF termination is often difficult to achieve even after ablation of an identified driver, as AF is generally maintained by multiple unstable driver domains, rather than a single stable source (12). This phenomenon becomes more prominent in longstanding AF, where extensive fibrosis and conduction heterogeneity provide a substrate for multiple reentries (13). Nonetheless, ablation of rotor and focal sources has been associated with reduction of late AF recurrence compared with trigger ablation alone (14). Accordingly, AF non-inducibility was used as the procedural endpoint in this study. The non-inducibility rate was 100% after in the *de novo* group, compared with 84.8% in the redo group (*p* = 0.350), likely reflecting baseline differences, as redo cases may be associated with more advanced atrial cardiomyopathy.

### 4.7 Correlation of focal sources with fractionation and peak frequency

Although long fractionated electrograms spanning more than 80% of the AF cycle length were frequently recorded at focal source sites, fractionation alone was not a reliable marker of focal activity (Supplementary Figure S12).

Fractionated electrograms recorded at focal sources may reflect components of reentrant circuits or localized driver activity sustaining AF. However, fractionation observed at other sites can arise from passive mechanisms, including delayed conduction, electrograms recorded across lines of conduction block, or wavefront collision. These regions are therefore likely bystander zones rather than critical substrates for AF maintenance (Supplementary Figure S7). Consequently, indiscriminate ablation guided solely by electrogram fractionation may be unnecessary and could expose patients to avoidable atrial injury.

In this study, no significant correlation was observed between ARV-identified focal sources and peak frequency during AF. High peak frequencies often reflect localized delayed conduction near the endocardial surface and do not necessarily indicate sites critical for AF perpetuation. Accordingly, we propose that targeting focal sources identified through ARV mapping represents a more rational and potentially more effective strategy than ablation approaches guided solely by electrogram fractionation or peak frequency analysis.

### 4.8 Clinical implications

Our findings suggest that ARV mapping before PVI has limited clinical utility, as it frequently reveals chaotic propagation patterns at this stage. The technique may be more valuable in two scenarios: (1) when PVI fails to suppress AF in *de novo* cases and (2) in redo procedures without pulmonary vein reconnection. With its high-resolution capability, ARV mapping may offer additional insights into AF mechanisms and patient-specific substrate characteristics. However, given the limited sample size and lack of long-term follow-up, these observations should be viewed as preliminary. Larger studies with extended follow-up are required to clarify the relationship between ARV patterns and clinical variables (e.g., AF duration, left atrial size) and to assess whether ARV mapping can aid in individualized prediction of AF recurrence.

## 5 Study limitations

This was a prospective, single-arm study conducted at a single center with a limited sample size, which may restrict generalizability. Adenosine triphosphate was not administered to induce AF, potentially limiting assessment of concealed triggers.

Although a standardized protocol was applied to ablate only ARV-identified focal sources, additional ablation was performed when AT or AF-triggering premature atrial contractions emerged. Thus, it cannot be conclusively established that the ARV-identified focal sources were the sole arrhythmogenic substrates. Potential operator bias in mapping interpretation and patient selection bias may also have influenced the findings. Finally, only acute outcomes in terms of AT/AF non-inducibility were evaluated; long-term follow-up is required to determine durability and clinical efficacy.

## 6 Conclusion

ARV mapping enables real-time visualization of AF activation patterns. In this study, ablation of ARV-identified focal sources effectively suppressed AF. This novel mapping approach provides mechanistic insights and may contribute to more individualized ablation strategies.

## Data Availability

The original contributions presented in the study are included in the article/Supplementary Material, further inquiries can be directed to the corresponding author.

## Supplementary material

The Supplementary Materialfor this article can be found online at: https://www.frontiersin.org/articles/10.3389/fcvm.2026.1719071/full#supplementary-material
