# Supplementary material for: Auto reference vector: a novel method for mapping atrial fibrillation
Source: Front Cardiovasc Med. 2026 Mar 2;13:1719071. doi: 10.3389/fcvm.2026.1719071 (PMC13006920; doi:10.3389/fcvm.2026.1719071)
Supplement: Supplementary file 1 [file Datasheet1.docx]

**SUPPLEMENTAL MATEERIAL**

Supplemental method..................................................................................................2

Table S1. All Patient characteristics............................................................................3

Table S2. Details of prior ablations.............................................................................5

Table S3. Mapping outcome........................................................................................7

Table S4. Characteristics of identified focal sources....................................................8

Figure S1. Score criteria for avoiding ventricular annotation......................................10

Figure S2. One-second fractionation score..................................................................11

Figure S3: Peak frequency and ARV............................................................................12

Figure S4. A de novo case with organized pattern and high reproducibility...............13

Figure S5. Passive activation pattern of right atrium..................................................13

Figure S6: Adjustment of sensitivity...........................................................................14

Figure S7: No correlation between fractionation and focal sources...........................15

Figure S8: Change of vector direction after ablation of focal sources........................16

Figure S9: Confirmation of block line during AF.......................................................17

Figure S10: Gap localization during AF.....................................................................18

Figure S11: Example of organized and disorganized pattern.....................................19

Figure S12: Example of electrograms at each site......................................................20

Supplemental VDO: Failure to annotate AF potential with auto sensitivity

**Supplemental Method**

To assess the relationship between focal sources and electrogram complexity, retrospective analysis was conducted using TurboMap. A one-second WOI was selected, and the fractionation score was calculated for this interval—referred to as the 1-s fractionation score (1FS). Regions with 1FS values exceeding 70% of the maximum 1FS in the chamber were further evaluated (Figure S2). Correlations with peak frequency values were also analyzed (Figure S3).

**Table S1** All Patient characteristics


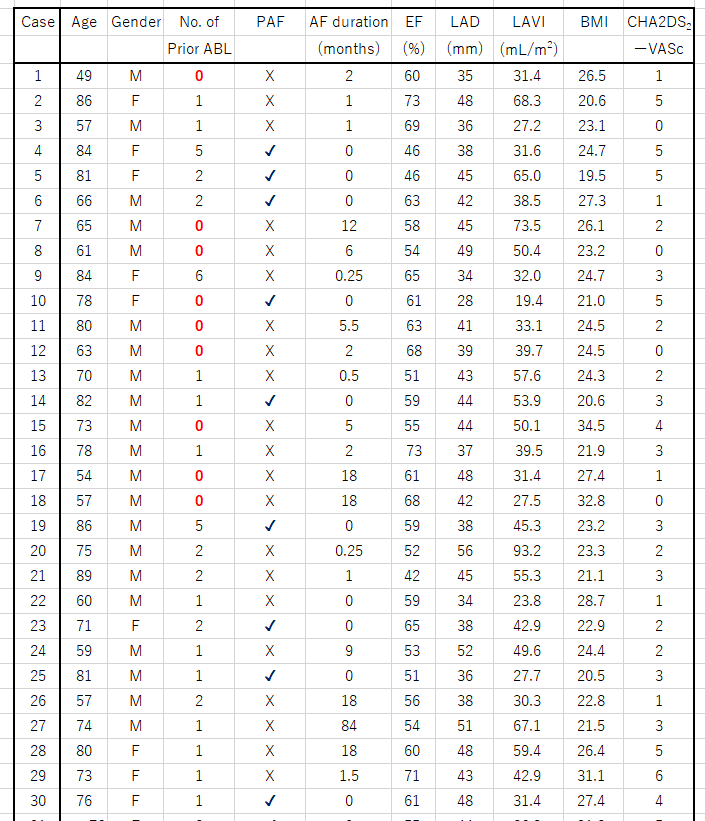


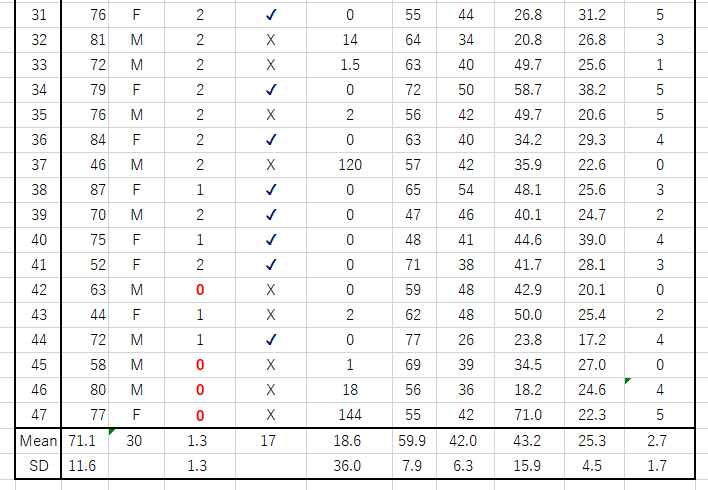


M= male; F= female; ABL= ablation;✓=yes; X= No

LAD= left atrial diameter; LAVI= left atrial volume index, BMI= body mass index

**Table S2** Details of Prior Ablations


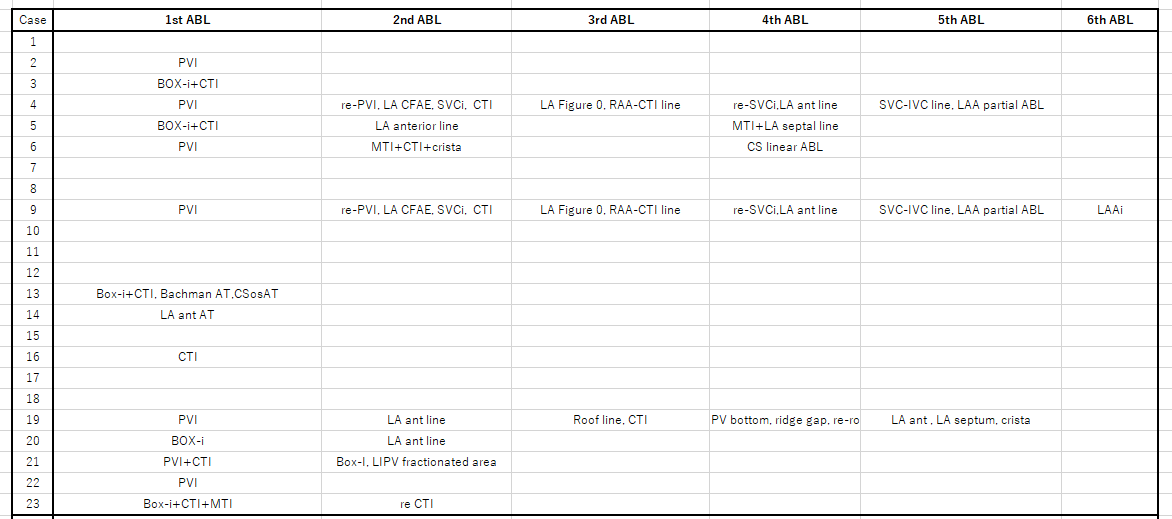


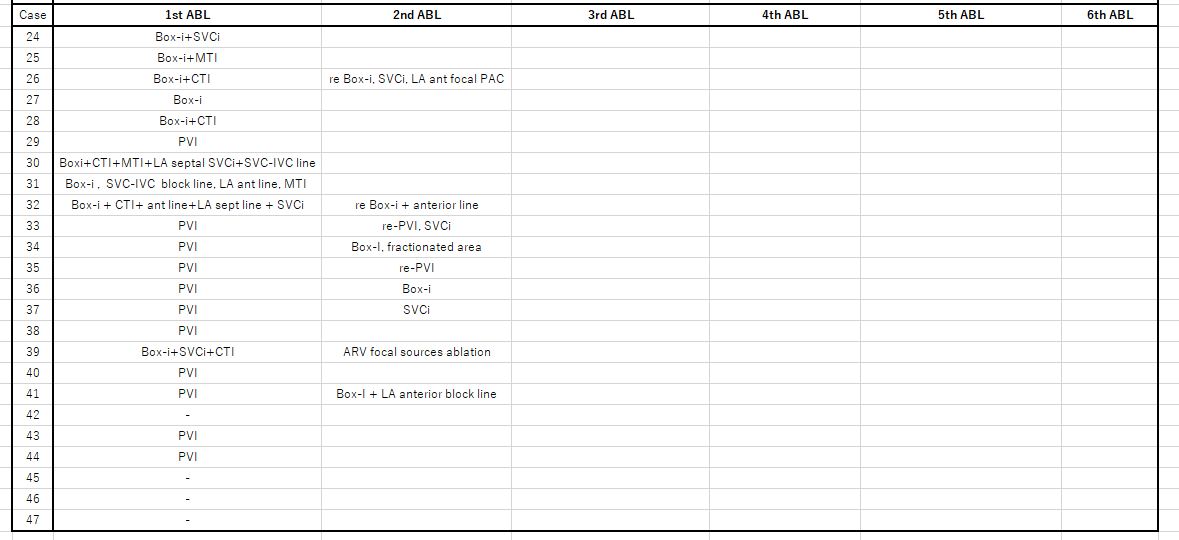


ABL= ablation; PVI=pulmonary vein isolation; Box-i= box isolation; CTI= cavotriscuspid isthmus ablation; SVCi= superior vena cava isolation

MTI = lateral mitral isthmus ablation; LA ant line = left atrial anterior linear ablation. CS os = coronary sinus ostium; LAAi= isolation of left atrial appendage

**Table S3:** Mapping parameters


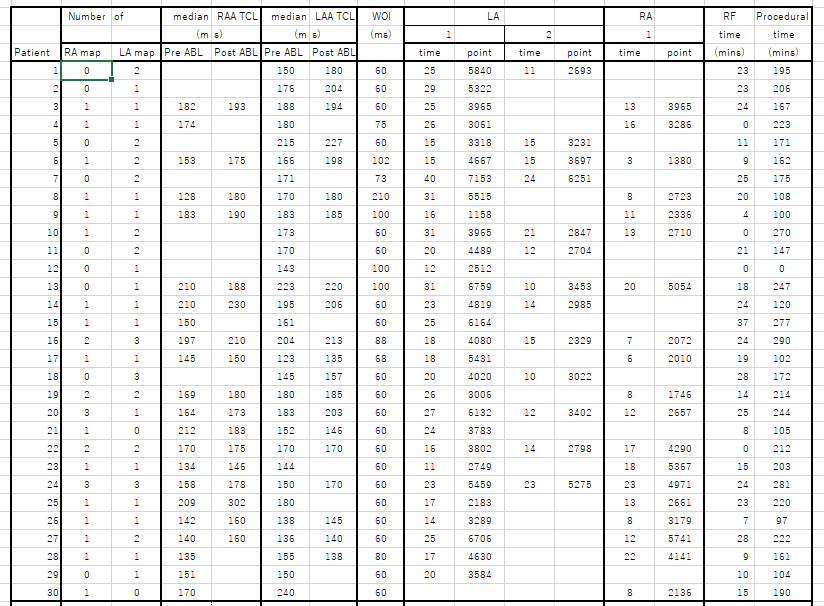


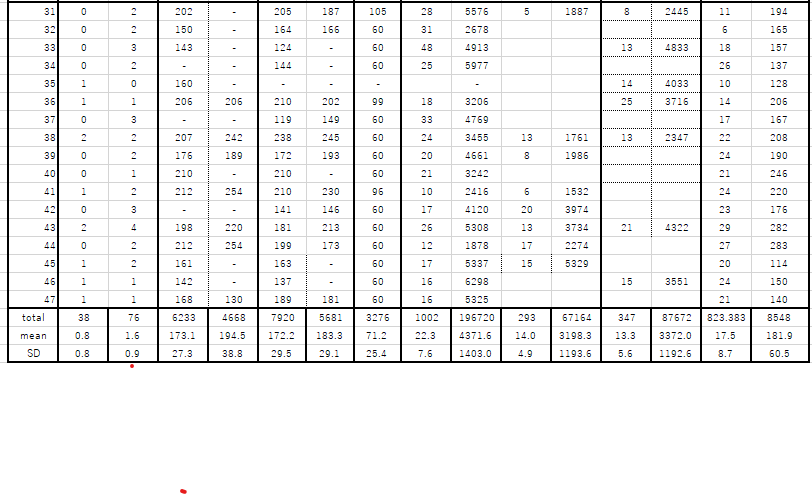


Time is expressed in minutes

RAA=right atrial appendage; LAA=left atrial appendage; TCL = tachycardia cycle length; WOI= window of Interest width

**Table S4**: Characteristics of identified focal sources


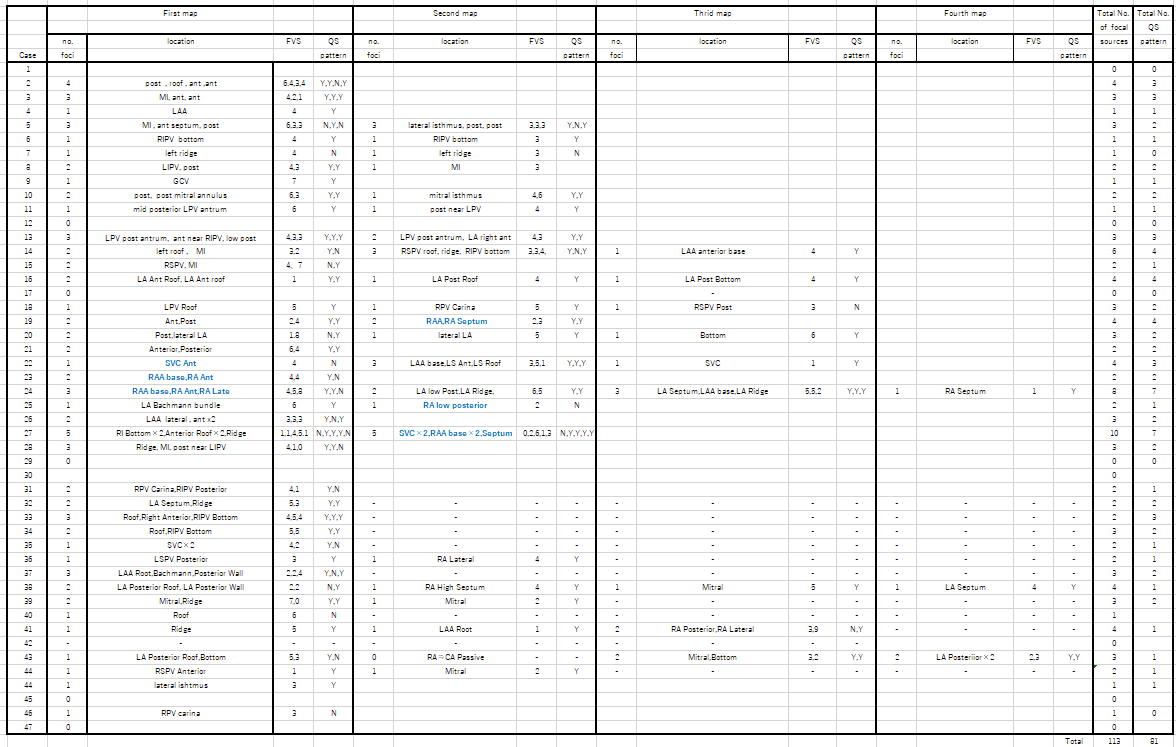


ARV maps were sequentially created after ablation of identified focal sources until AT/AF non-inducibility was obtained except in case 1,5,6,7,8,10,11,13,18,20,42 and 45 where repeat maps were obtained without ablation to confirm reproducibility.

Blue color indicates focal sources located in the right atrium.

**Abbreviations:** FVS = focal vector score; ant = left atrial anterior wall; post = left atrial posterior wall; MI = mitral isthmus; LAA = left atrial appendage; RAA = right atrial appendage; GCV = great cardiac vein; LPV = left pulmonary veins; LIPV = left inferior pulmonary vein; RIPV = right inferior pulmonary vein.

**A**

**
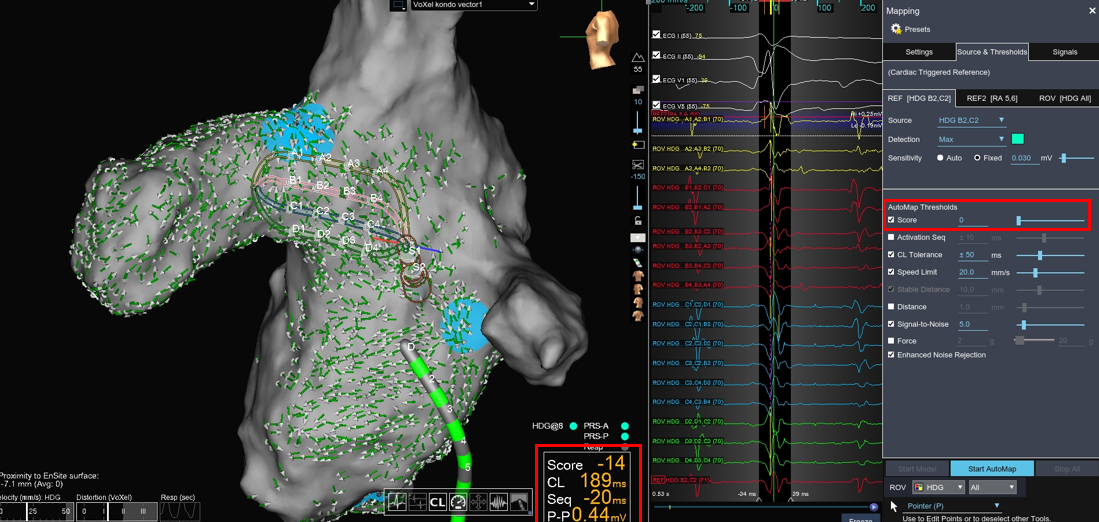
**

**B
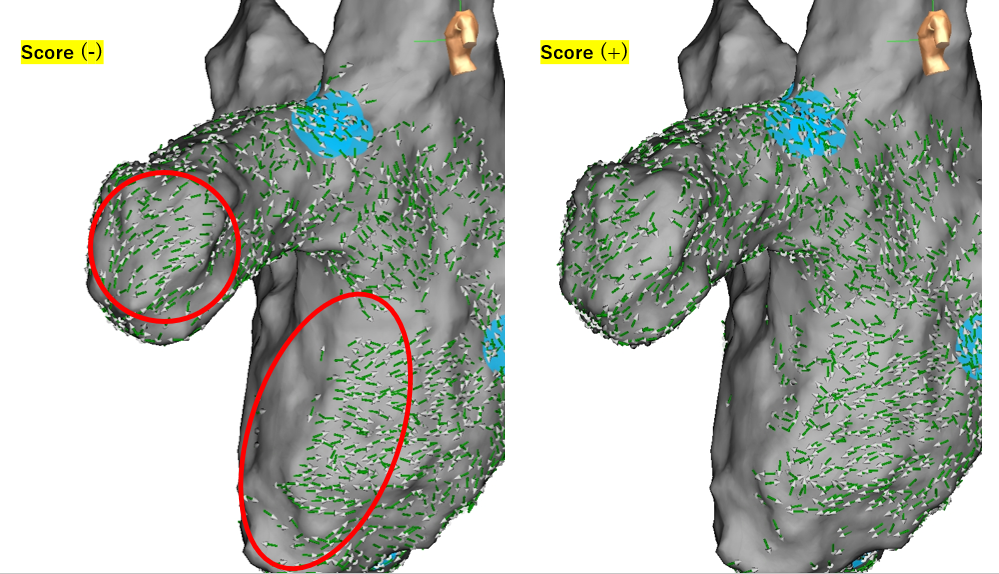
**

**Figure S 1:**

**(A)** Annotation at ventricular potential was avoided by applying a score threshold > 0.

**(B)** Near the LAA and mitral annulus, vector direction changed from ventricular to atrial after applying the score criterion. Red circles indicate regions with ventricular vectors; blue dots indicate identified focal sources.

**
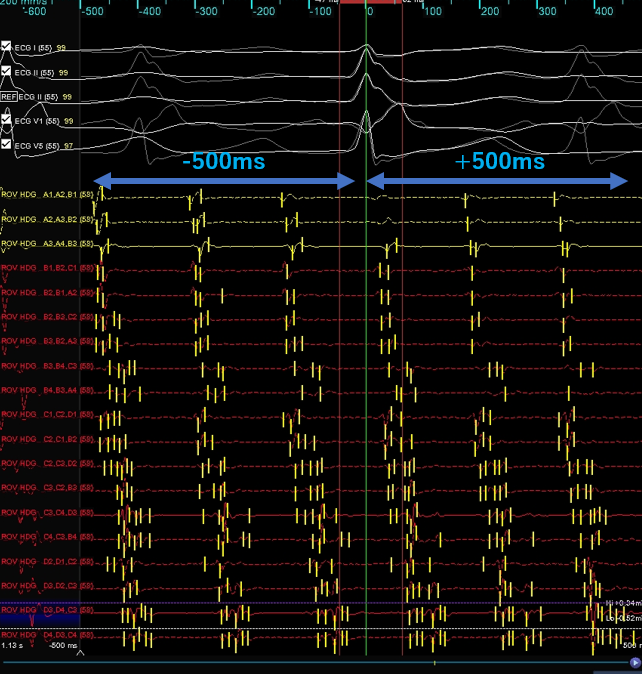
**

**B**


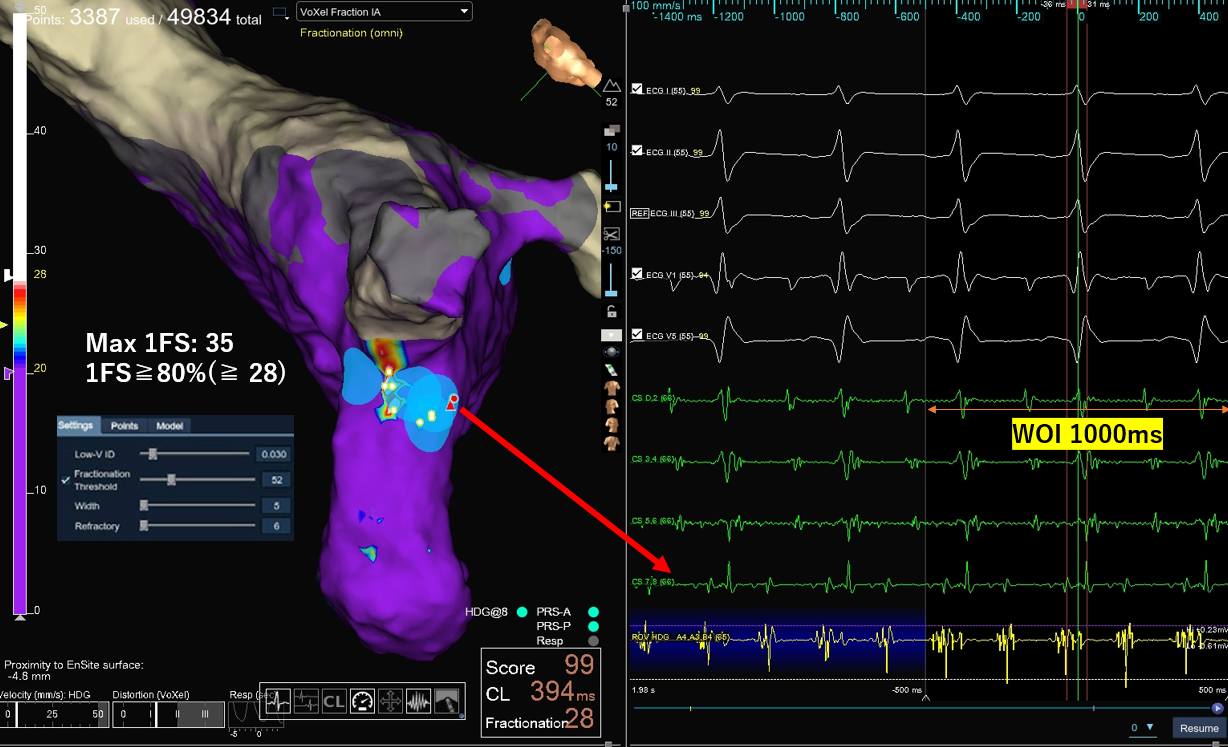


**Figure S 2**

**(A)** Fractionated potentials during AF were evaluated by Turbo map with a window of interest set to 1 second. The time at which any potential exceeding the prespecified noise crossed the baseline with in this interval was defined as the *one-second fractionation score* (1FS). Other settings include Low-V ID= 0.03, Width = 5 (nominal 12), Refractory = 6 (nominal 30).

**(B)** Representative case showing concordance between ARV and fractionation (case 4). The potential indicated by the red arrow corresponds to yellow dots, which mark sites with a 1FS greater than 80% (≥28) of the maximal 1FS (35) in this patient. Blue dots denote focal sources identified by ARV.

**A**


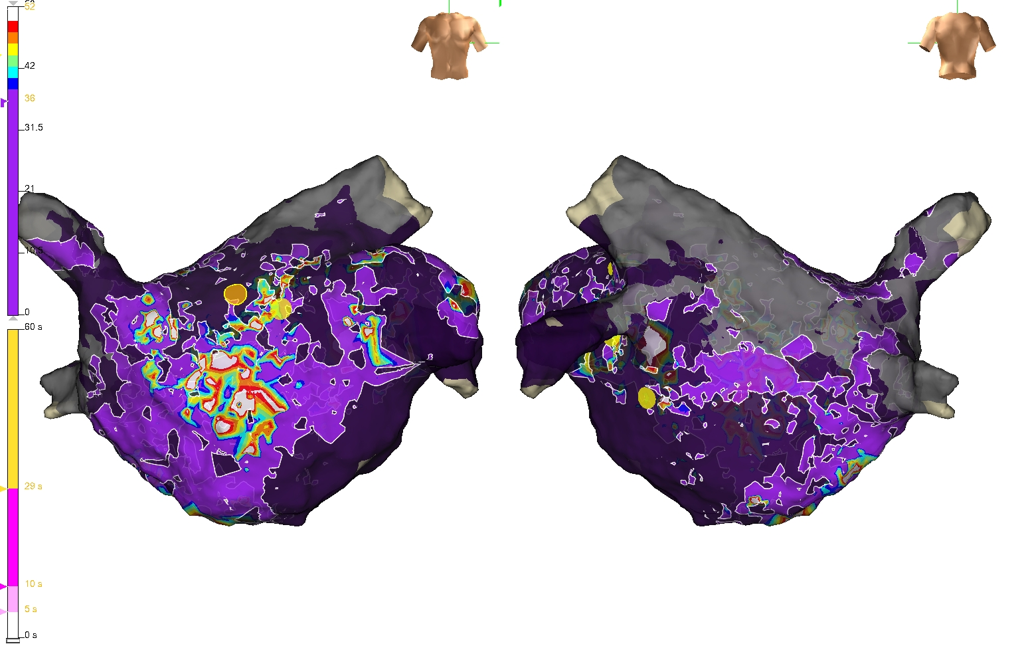


**B**


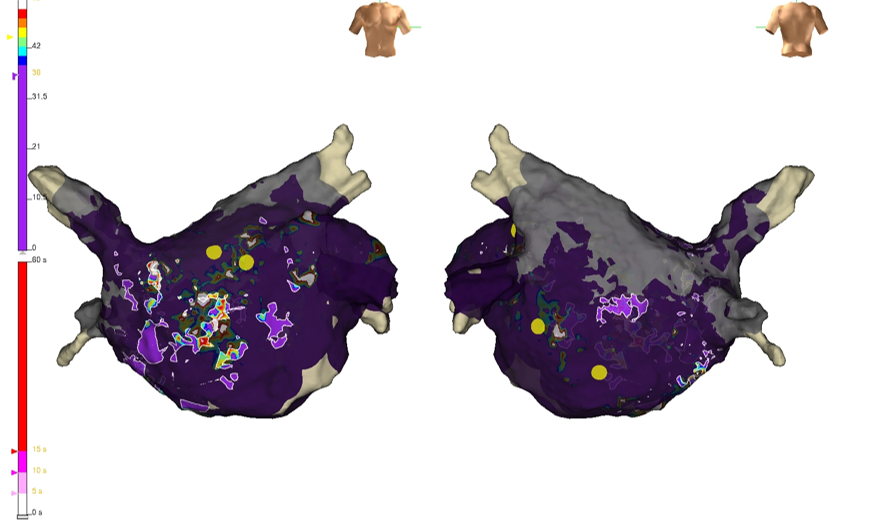


**Figure S 3**

Emphasis map on one-second fractionation score for (A) peak frequency (PF) > 350Hz and (B)PF > 500Hz. Fractionation colocalized with are high PF area but not with focal sources identified by ARV (yellow and orange circles)


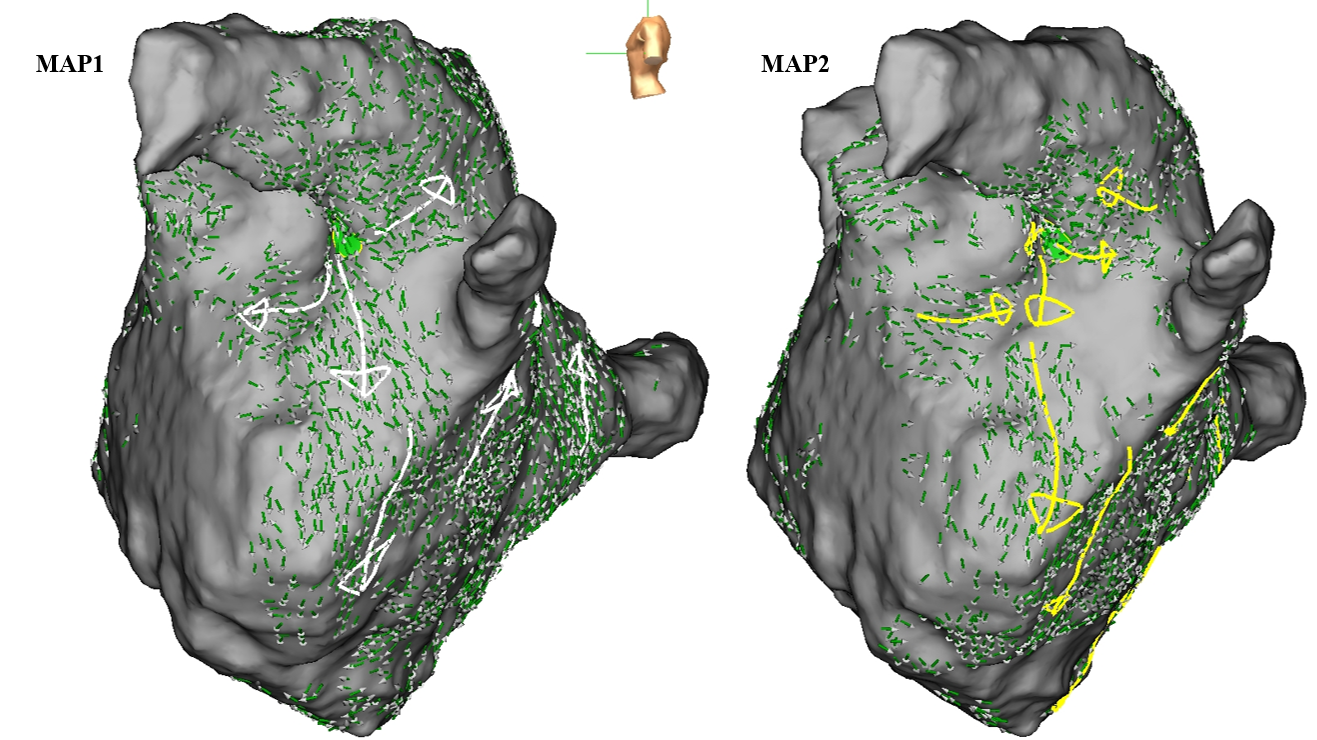


**Figure S 4**: Despite being a *de novo* case (case 7) two consecutive maps demonstrated an organized pattern with high reproducibility. A focal source was identified at the left atrial ridge, and inclusion of this site within left pulmonary vein isolation line that rendered AF non-inducible.


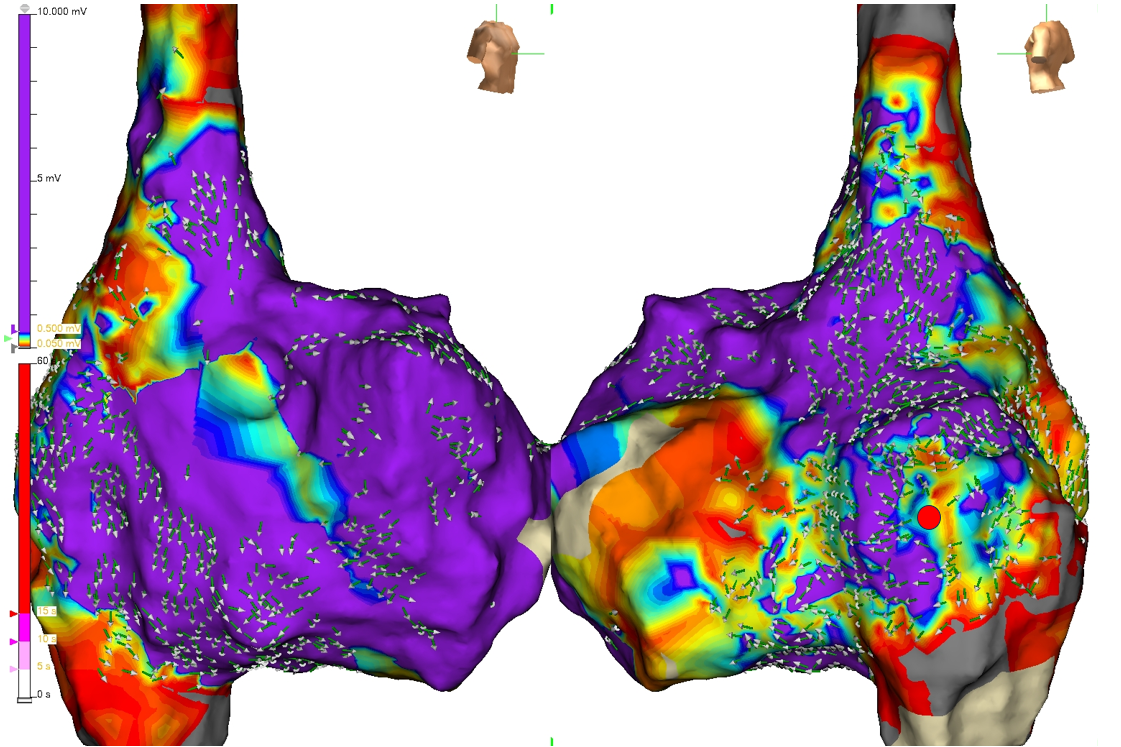


**Figure S 5**: Representative Case (Case 9) of focal activation pattern near low septum of right atrium (red circle). We can readily differentiate between RA septal focal sources and passive activation by mapping of LA. RA was passively activated in this case.


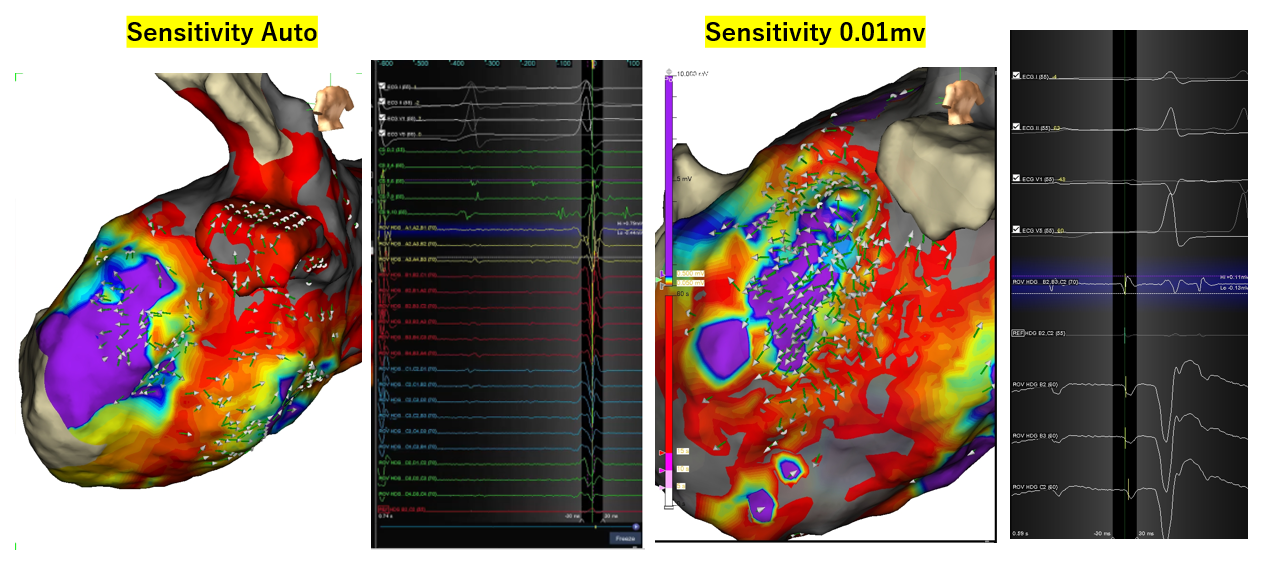


**Figure S 6: Adjustment of sensitivity**

In Case 9, the sensitivity of the reference electrodes needed adjustment to ensure accurate ARV display. When the sensitivity was initially set to auto, NAVx system failed to annotate the minimal atrial potentials near lateral mitral annulus, instead annotating larger ventricular potentials. As a result, those points were rejected based on the scoring criteria. After manually adjusting sensitivity to 0.01mV, a focal source near mitral annulus was successfully identified.


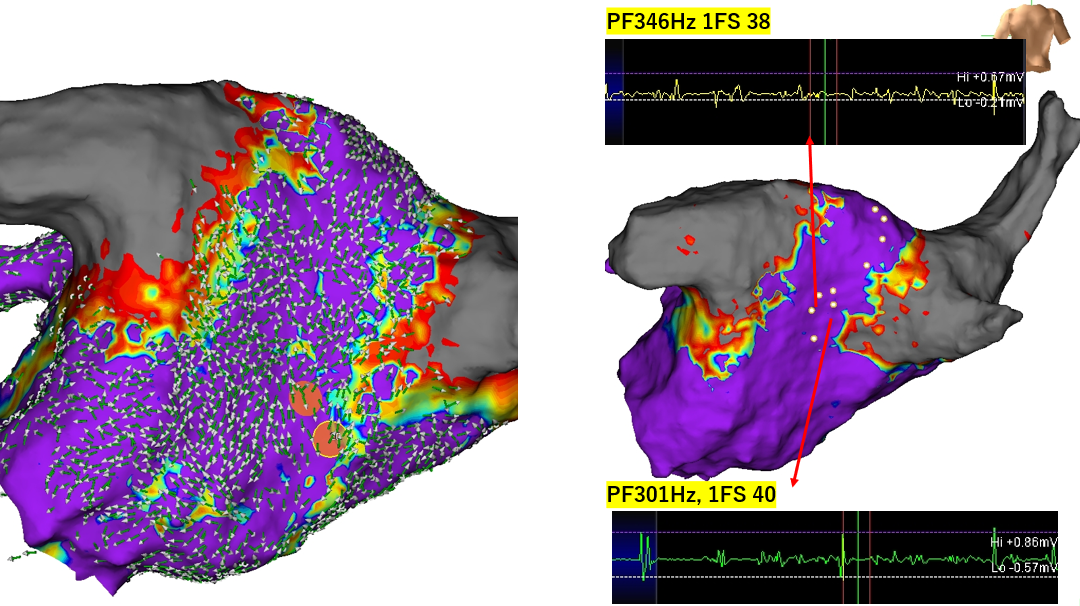


**Figure S 7: No correlation was observed between electrogram fractionation and focal sources in most cases.**

In Case 6, long fractionated potentials (1FS of 38-40) were recorded at LA posterior wall. However, ARV mapping indicated that the posterior wall was passively activated, with vectors colliding at the mid-portion. PF = peak frequency; 1FS= one-second fractionation score


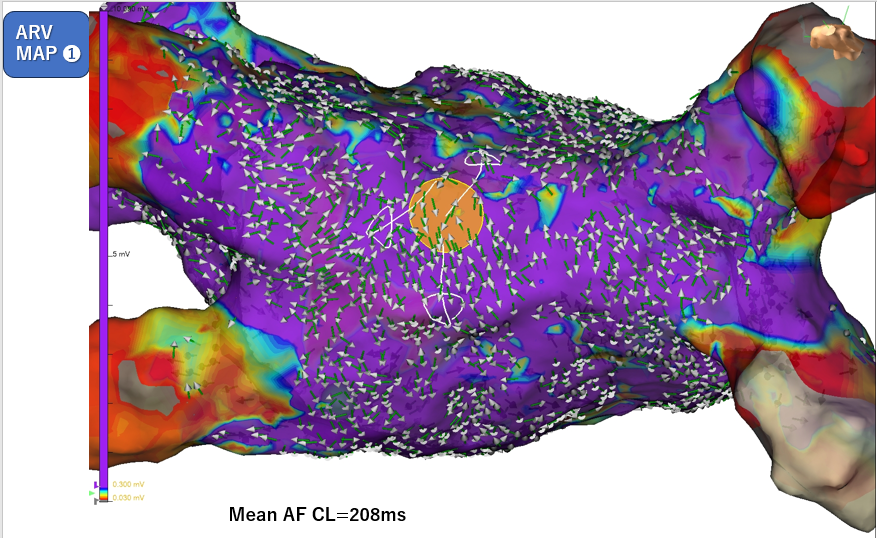


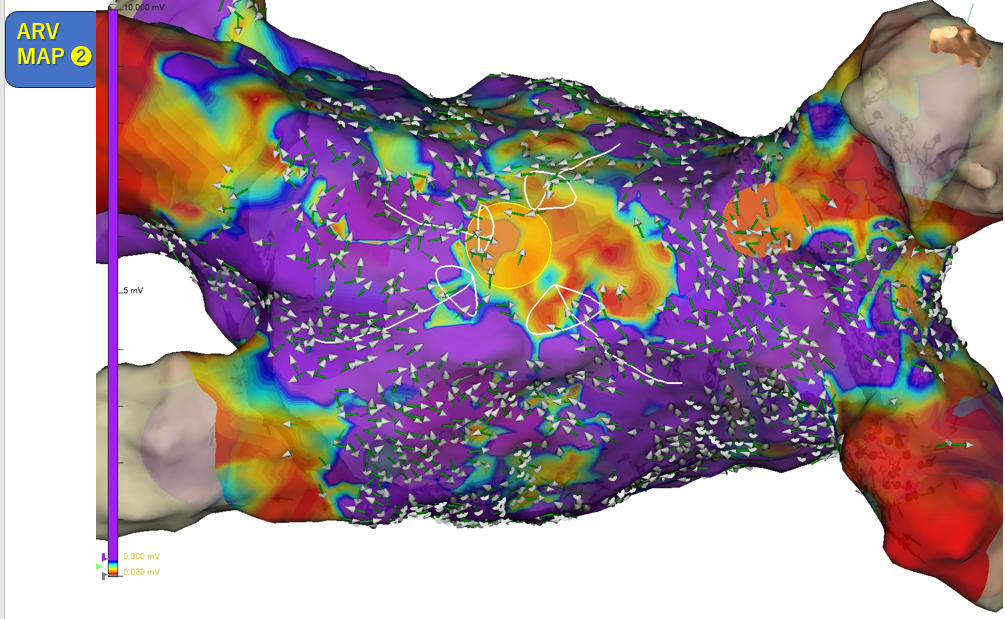
**Figure S 8:** **Repeated ARV mapping after ablation of a focal source at LA roof**

Following cardioversion and reinduction of AF, second ARV map demonstrated that the previously active area at the LA roof became passively activated, while a new prominent source emerged near the right superior pulmonary vein.


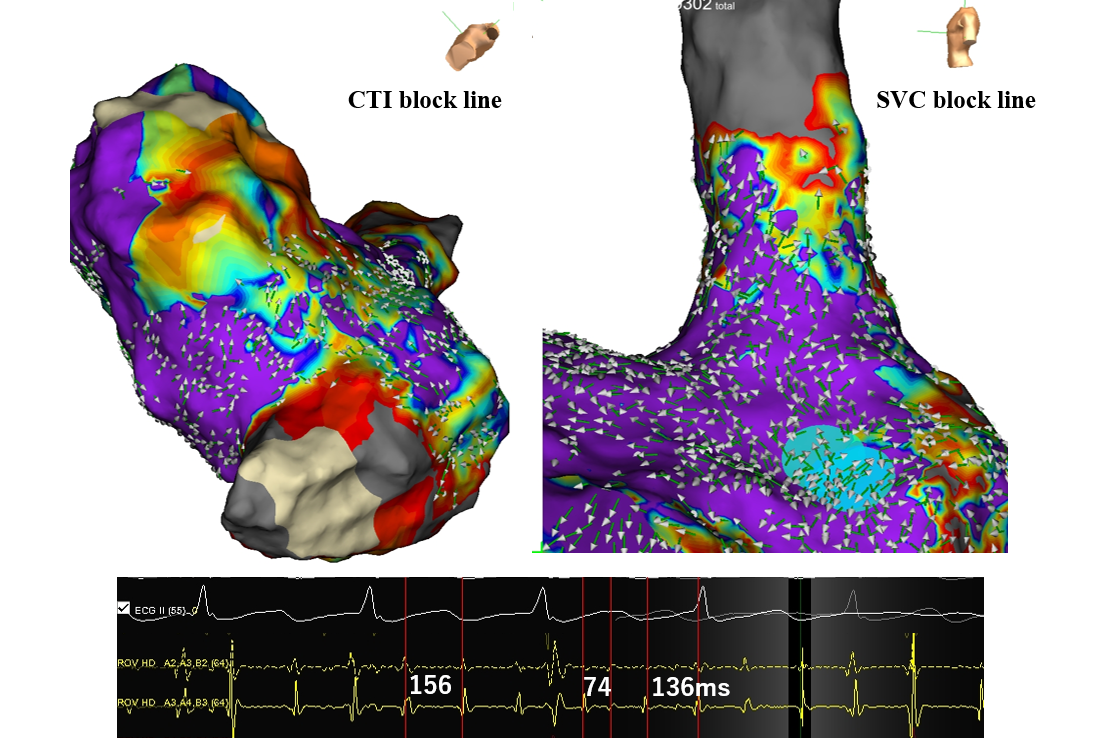


**Figure S 9: Confirmation of conduction block lines during AF**

ARV mapping during AF demonstrates vector collisions along lines of block or vectors terminated at conduction dead ends, indicating areas of impaired conduction.

**A**

**
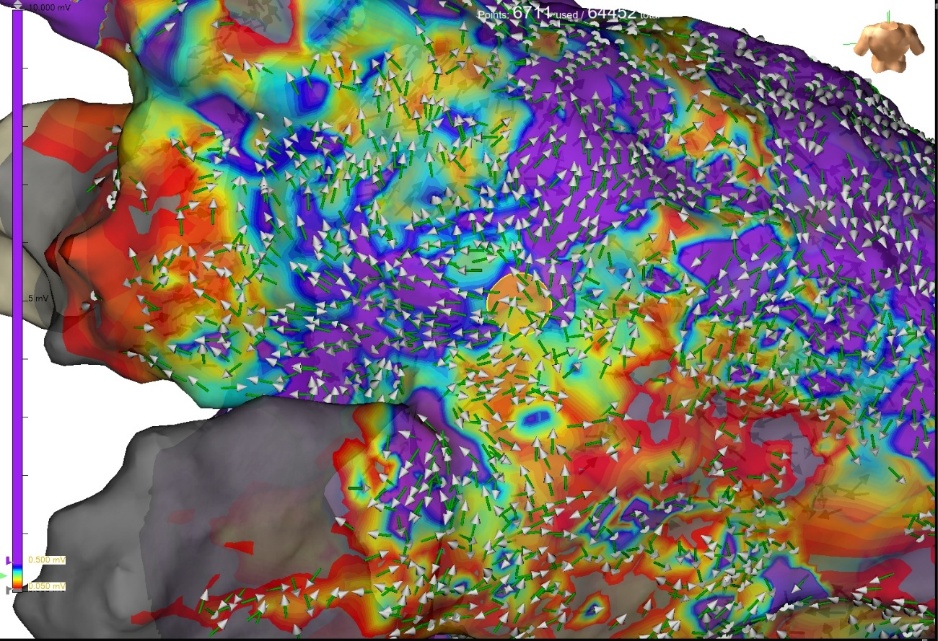
**

**B**

**
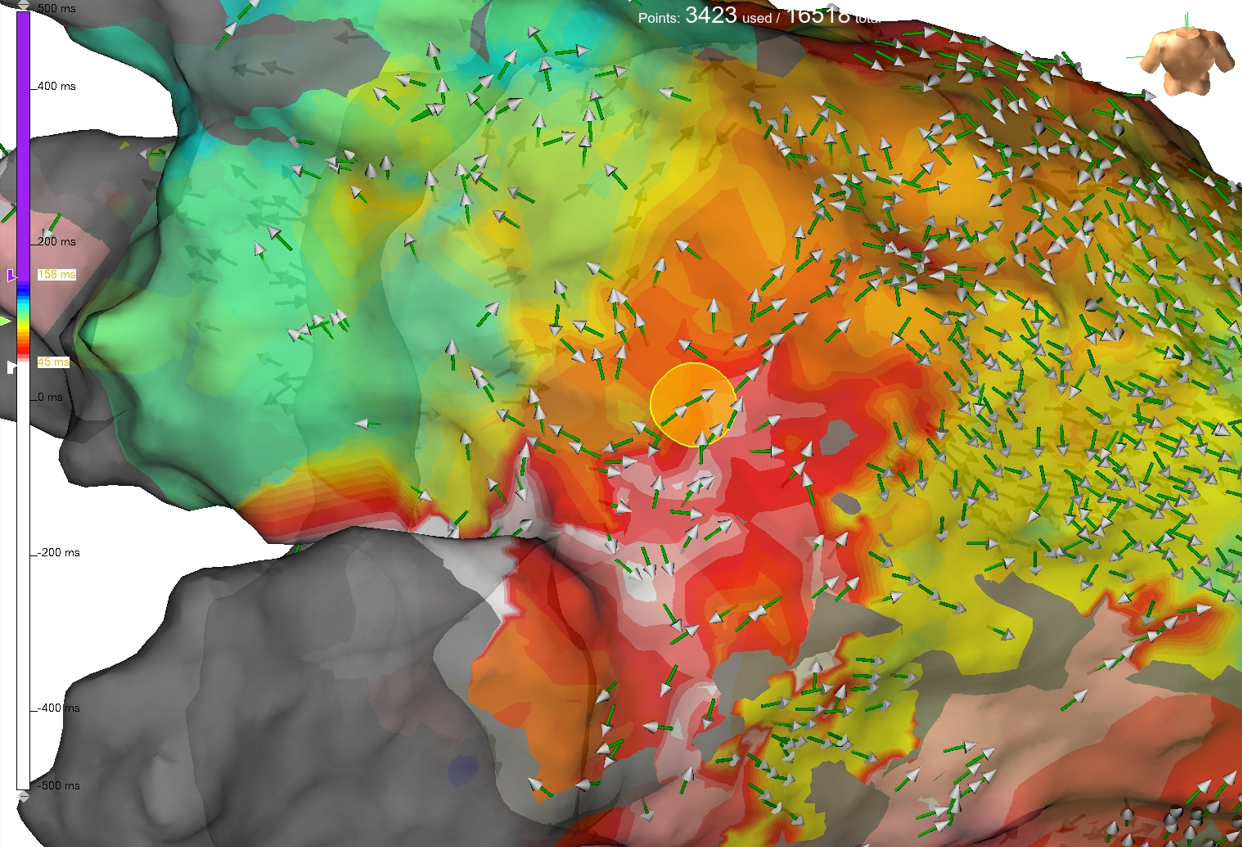
**

**Figure S 10: Gap localization during AF**

(A) In Case13, the patient had reconnection of left pulmonary veins following pulsed field ablation performed 3 months earlier. ARV mapping during AF localized the conduction gap at the posterior antrum with high resolution

(B) The gap location (orange circle) was confirmed by cardioversion followed by pacing from the distal coronary sinus.

**A**


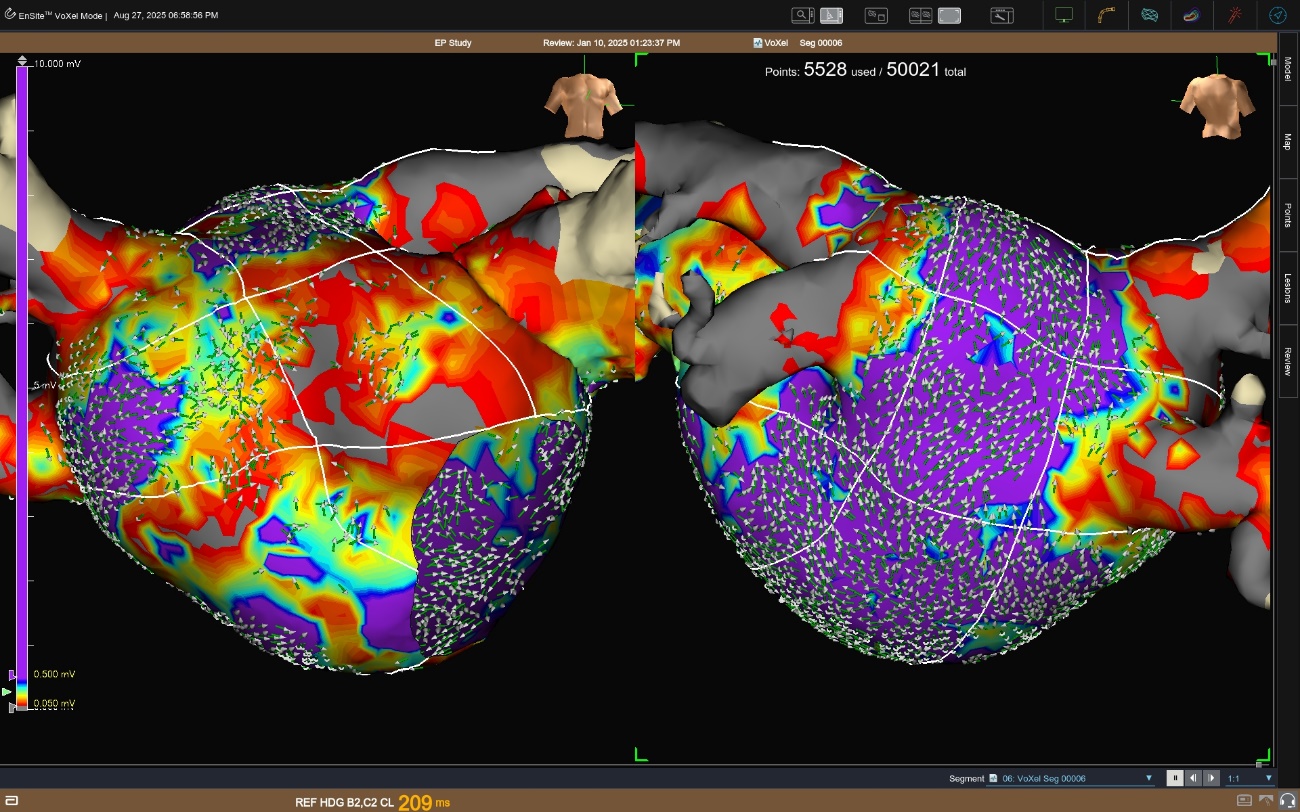


**B**

**
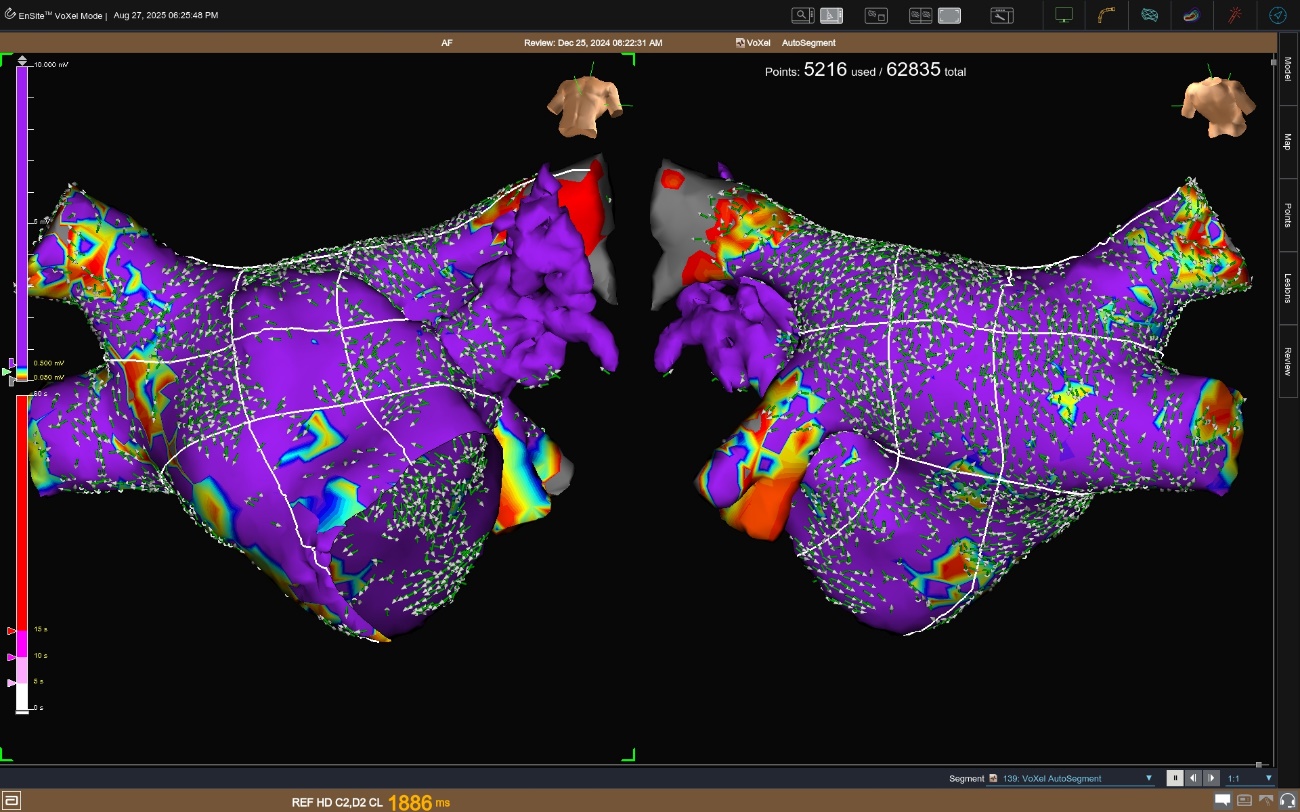
**

**Figure S11: Example of organized and disorganized LA activation pattern**

The anterior and posterior aspect of LA each were divided in to 9 segments.

The concordance of vectors within each segment was evaluated and the segment was considered *organized* if >70% of adjacent vectors pointed to the same direction. The entire chamber was classified as *organized* if >70%, i.e., more than 13 out of 18 were organized. Only regions with an omnipolar voltage >0.50mV were included in the analysis.

1. A redo case showing an organized pattern, with 16 out of 18 segments classified as organized.
2. A de novo case showing a disorganized pattern, with 0 out of 18 segments classified as organized.


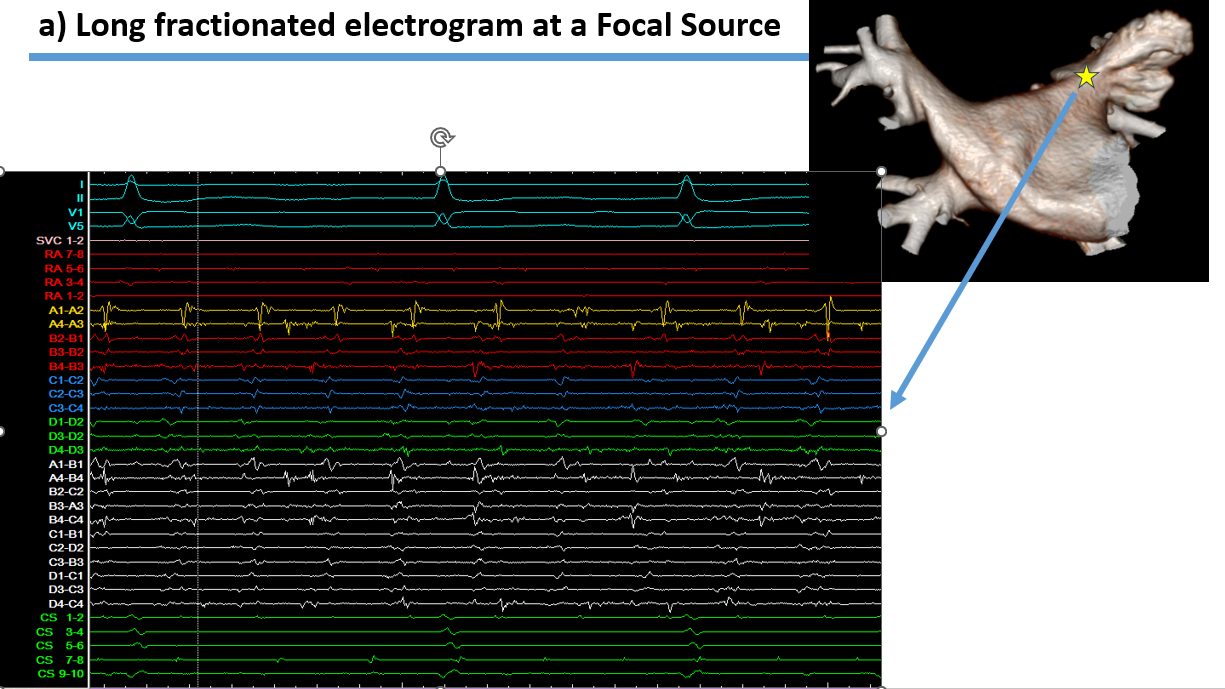


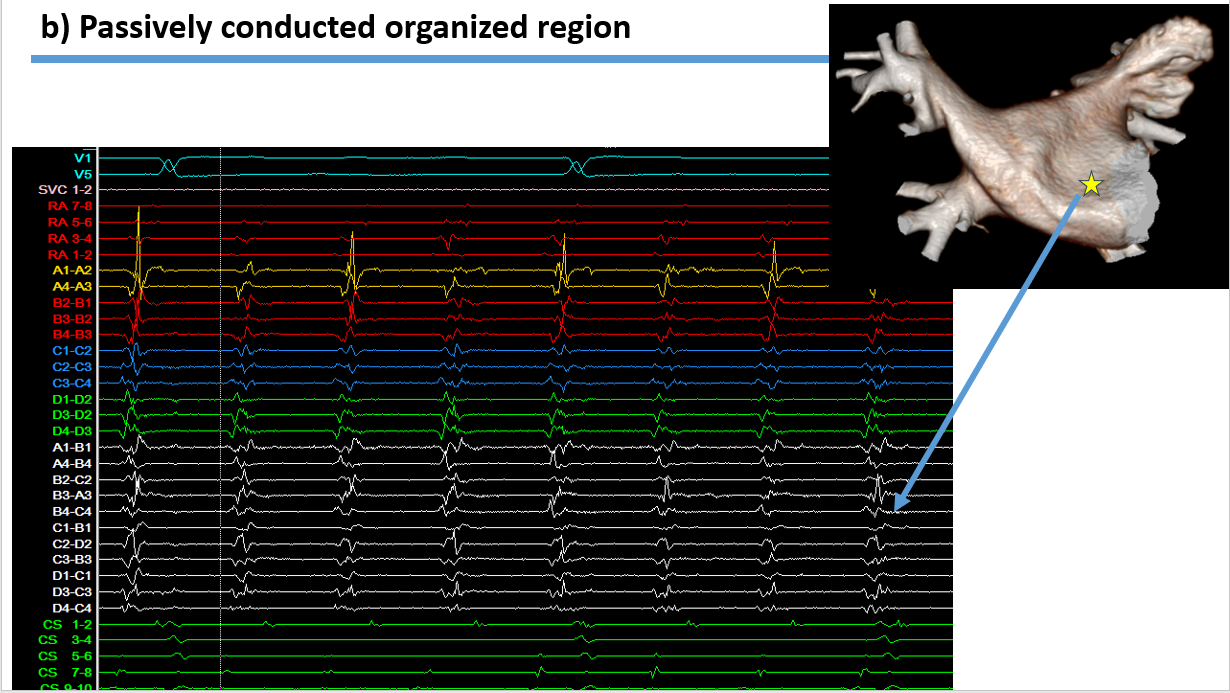


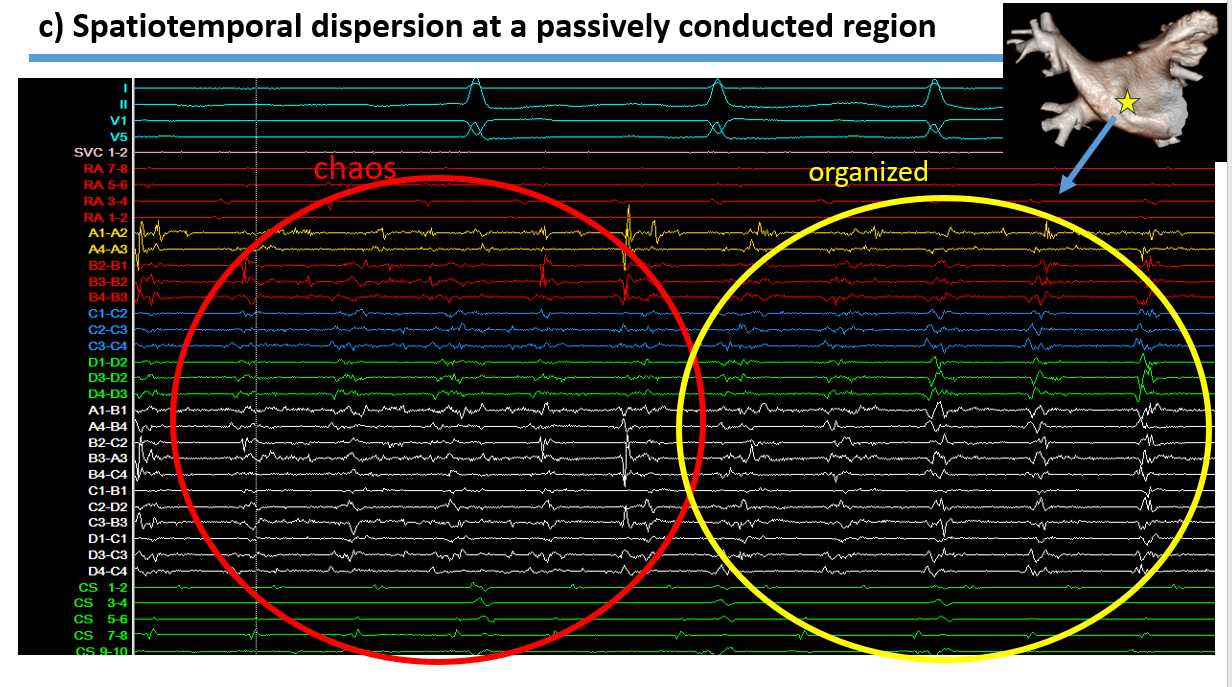


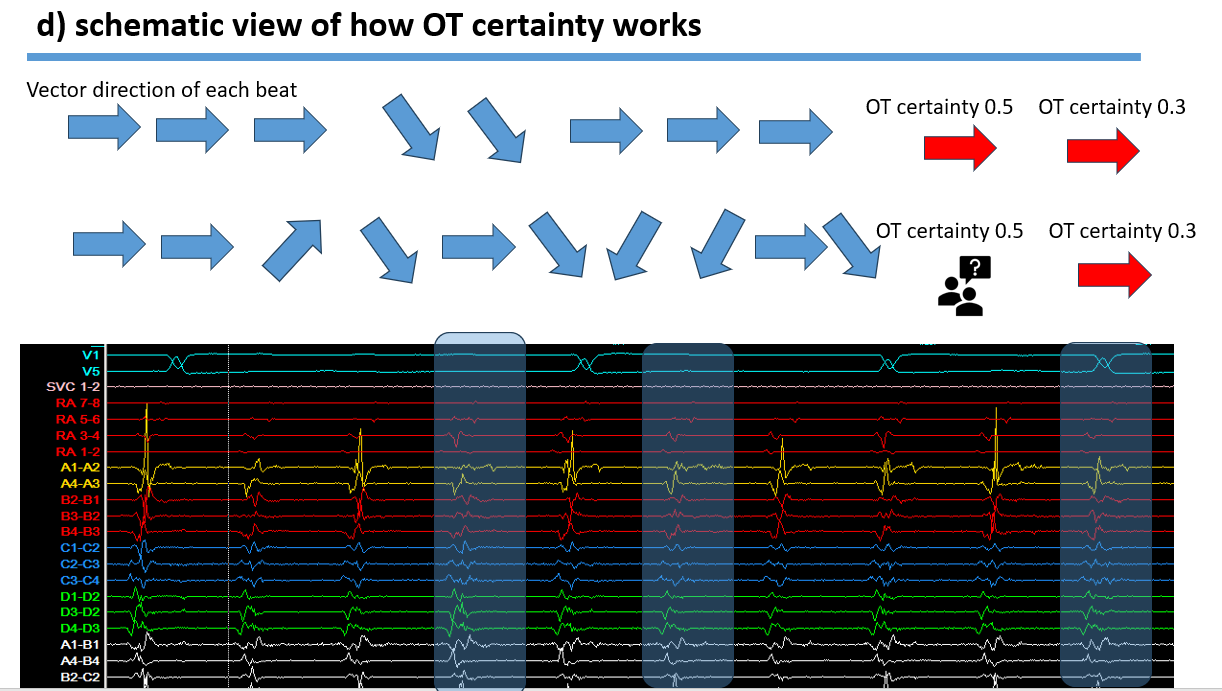


Figure S12) Representative electrograms from each mapping site and a schematic depiction of Omnipolar Technology (OT) certainty. Regions exhibiting spatiotemporal dispersion demonstrate reduced vector concordance. Application of an OT certainty threshold of 0.5 selectively retains activation vectors with high directional consistency on the Auto-Reference Vector (ARV) map.
